# Supplementary figures and images for: Increased burden of cardiovascular disease in people with liver disease: unequal geographical variations, risk factors and excess years of life lost
Source: J Transl Med. 2022 Jan 3;20:2. doi: 10.1186/s12967-021-03210-9 (PMC8722174; doi:10.1186/s12967-021-03210-9)

Additional file 1. Comorbidity patterns in patients with liver disease separated by sex and age.

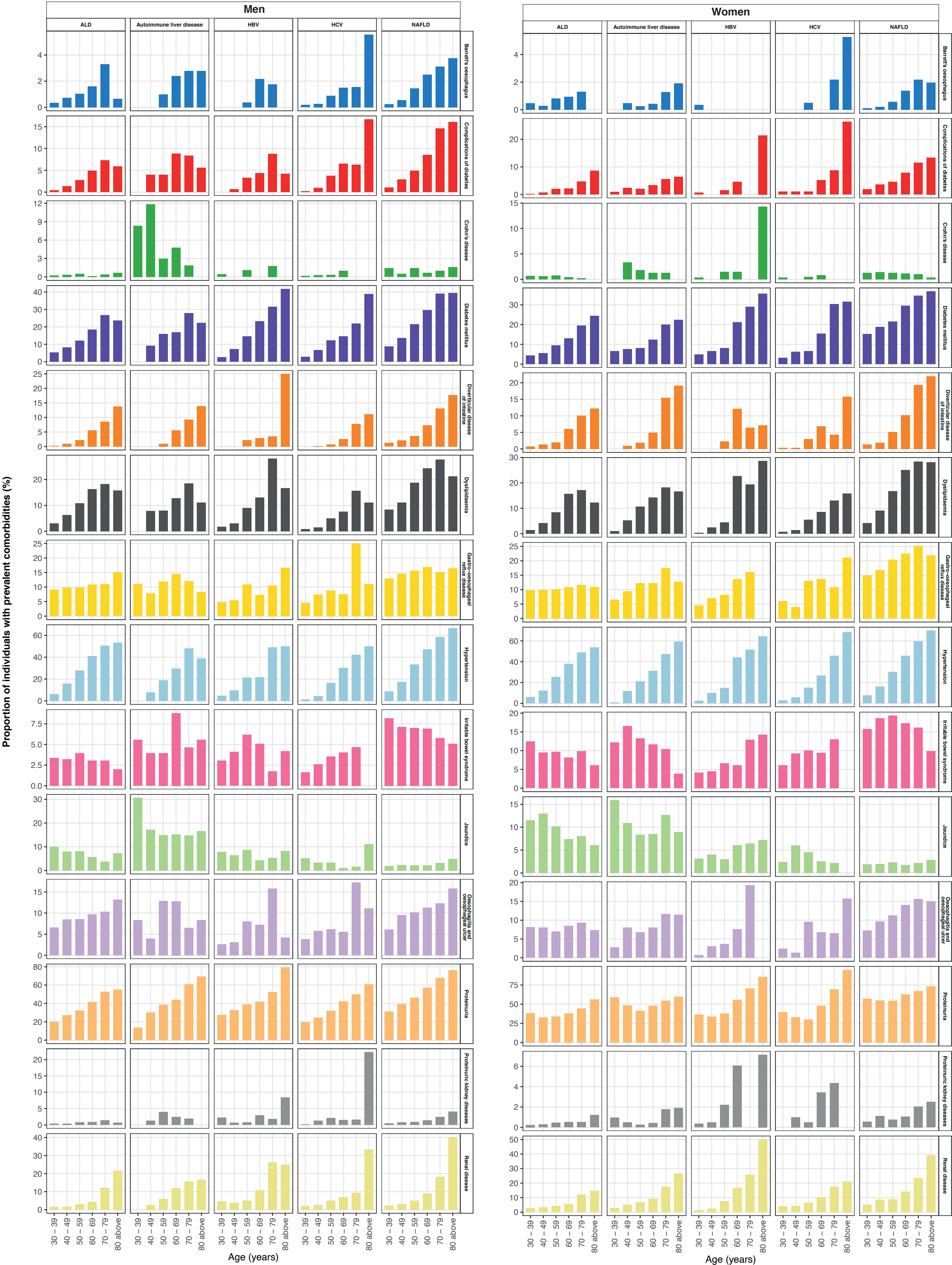

Supplement: Supplementary file 1 — Additional file 1: Comorbidity patterns in patients with liver disease separated by sex and age. [file 12967_2021_3210_MOESM1_ESM.pdf]
