# Supplementary material for: Increased burden of cardiovascular disease in people with liver disease: unequal geographical variations, risk factors and excess years of life lost
Source: J Transl Med. 2022 Jan 3;20:2. doi: 10.1186/s12967-021-03210-9 (PMC8722174; doi:10.1186/s12967-021-03210-9)

Additional file 2. Proportion of patients within each age-sex stratum experiencing incident CVD (first presentation) are shown for (A) each of the 17 individual CVDs. (B) The 17 conditions are grouped into five categories and proportions are shown for each category.

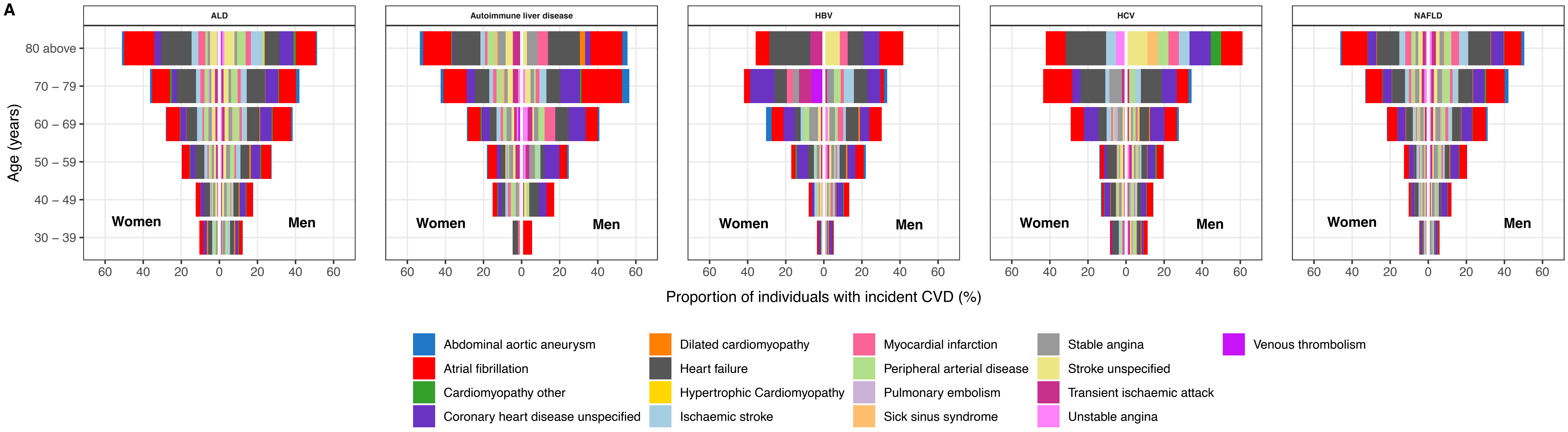

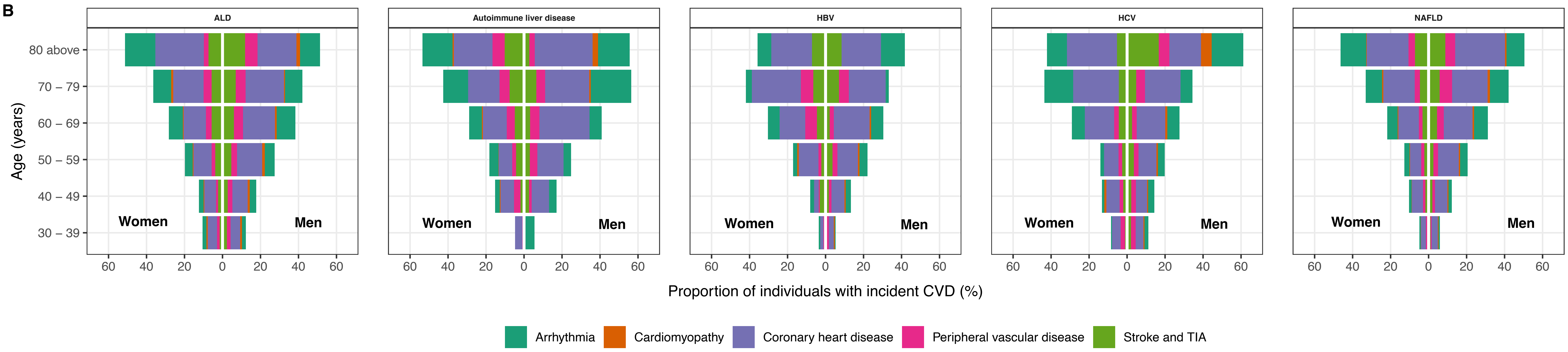

Supplement: Supplementary file 2 — Additional file 2: Proportion of patients within each age–sex stratum experiencing incident CVD (first presentation) are shown for A each of the 17 individual CVDs. B The 17 conditions are grouped into five categories and proportions are shown for each category. [file 12967_2021_3210_MOESM2_ESM.pdf]
