# Supplementary material for: Increased burden of cardiovascular disease in people with liver disease: unequal geographical variations, risk factors and excess years of life lost
Source: J Transl Med. 2022 Jan 3;20:2. doi: 10.1186/s12967-021-03210-9 (PMC8722174; doi:10.1186/s12967-021-03210-9)

Additional file 3. Excess years life lost (YLL) attributable to CVD in patients with liver disease separated by sex.

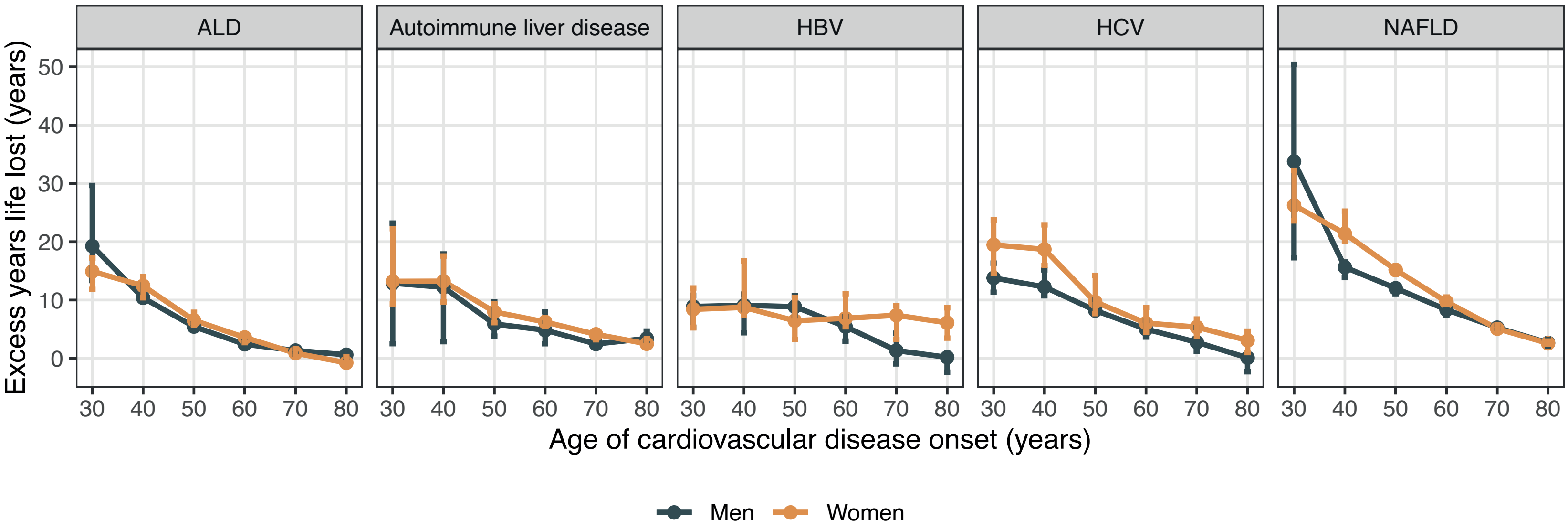

Supplement: Supplementary file 3 — Additional file 3: Excess years life lost (YLL) attributable to CVD in patients with liver disease separated by sex. [file 12967_2021_3210_MOESM3_ESM.pdf]
