# Supplementary material for: Increased burden of cardiovascular disease in people with liver disease: unequal geographical variations, risk factors and excess years of life lost
Source: J Transl Med. 2022 Jan 3;20:2. doi: 10.1186/s12967-021-03210-9 (PMC8722174; doi:10.1186/s12967-021-03210-9)
Supplement: Supplementary file 4 — Additional file 4: Baseline characteristics for patients with liver disease. [file 12967_2021_3210_MOESM4_ESM.pdf]

Additional file 4. Baseline characteristics for patients with liver disease

	ALD			Autoimmune liver disease			HBV			HCV			NAFLD		
	Men	Women	Overall	Men	Women	Overall	Men	Women	Overall	Men	Women	Overall	Men	Women	Overall
**n**	8560	4285	12845	482	1728	2210	1019	734	1753	2135	977	3112	10751	10177	20928
**Practice region (%)**															
**North East**	285 (3.3)	141 (3.3)	426 (3.3)	12 (2.5)	65 (3.8)	77 (3.5)	17 (1.7)	12 (1.6)	29 (1.7)	52 (2.4)	24 (2.5)	76 (2.4)	301 (2.8)	318 (3.1)	619 (3.0)
**North West**	1929 (22.5)	1007 (23.5)	2936 (22.9)	87 (18.0)	343 (19.8)	430 (19.5)	199 (19.5)	123 (16.8)	322 (18.4)	537 (25.2)	214 (21.9)	751 (24.1)	2238 (20.8)	2045 (20.1)	4283 (20.5)
**Yorkshire & The Humber**	374 (4.4)	200 (4.7)	574 (4.5)	16 (3.3)	92 (5.3)	108 (4.9)	26 (2.6)	21 (2.9)	47 (2.7)	81 (3.8)	32 (3.3)	113 (3.6)	386 (3.6)	358 (3.5)	744 (3.6)
**East Midlands**	327 (3.8)	174 (4.1)	501 (3.9)	23 (4.8)	66 (3.8)	89 (4.0)	26 (2.6)	27 (3.7)	53 (3.0)	69 (3.2)	40 (4.1)	109 (3.5)	306 (2.8)	292 (2.9)	598 (2.9)
**West Midlands**	1065 (12.4)	550 (12.8)	1615 (12.6)	47 (9.8)	188 (10.9)	235 (10.6)	87 (8.5)	79 (10.8)	166 (9.5)	162 (7.6)	79 (8.1)	241 (7.7)	992 (9.2)	946 (9.3)	1938 (9.3)
**East of England**	758 (8.9)	390 (9.1)	1148 (8.9)	54 (11.2)	182 (10.5)	236 (10.7)	84 (8.2)	61 (8.3)	145 (8.3)	206 (9.6)	104 (10.6)	310 (10.0)	1010 (9.4)	966 (9.5)	1976 (9.4)
**South West**	1074 (12.5)	517 (12.1)	1591 (12.4)	65 (13.5)	218 (12.6)	283 (12.8)	118 (11.6)	70 (9.5)	188 (10.7)	299 (14.0)	147 (15.0)	446 (14.3)	1241 (11.5)	1239 (12.2)	2480 (11.9)
**South Central**	897 (10.5)	436 (10.2)	1333 (10.4)	62 (12.9)	177 (10.2)	239 (10.8)	90 (8.8)	72 (9.8)	162 (9.2)	224 (10.5)	111 (11.4)	335 (10.8)	1112 (10.3)	1040 (10.2)	2152 (10.3)
**London**	979 (11.4)	411 (9.6)	1390 (10.8)	48 (10.0)	175 (10.1)	223 (10.1)	283 (27.8)	202 (27.5)	485 (27.7)	337 (15.8)	149 (15.3)	486 (15.6)	1762 (16.4)	1599 (15.7)	3361 (16.1)
**South East Coast**	872 (10.2)	459 (10.7)	1331 (10.4)	68 (14.1)	222 (12.8)	290 (13.1)	89 (8.7)	67 (9.1)	156 (8.9)	168 (7.9)	77 (7.9)	245 (7.9)	1403 (13.0)	1374 (13.5)	2777 (13.3)
**Age (years, median [IQR])**	54.92 [46.71, 63.26]	54.52 [46.54, 62.95]	54.83 [46.63, 63.14]	62.47 [50.49, 71.72]	63.49 [53.41, 72.93]	63.29 [53.06, 72.59]	49.60 [41.05, 58.63]	43.14 [36.45, 54.33]	47.10 [38.32, 57.00]	46.75 [39.70, 54.75]	46.09 [38.51, 55.92]	46.50 [39.29, 54.96]	55.80 [46.58, 65.67]	57.76 [48.92, 67.55]	56.79 [47.77, 66.56]
**Age categories (%)**															
30 - 39	886 (10.4)	418 (9.8)	1304 (10.2)	36 (7.5)	107 (6.2)	143 (6.5)	231 (22.7)	288 (39.2)	519 (29.6)	560 (26.2)	294 (30.1)	854 (27.4)	1196 (11.1)	912 (9.0)	2108 (10.1)
40 - 49	2053 (24.0)	1093 (25.5)	3146 (24.5)	76 (15.8)	211 (12.2)	287 (13.0)	293 (28.8)	200 (27.2)	493 (28.1)	727 (34.1)	302 (30.9)	1029 (33.1)	2415 (22.5)	1869 (18.4)	4284 (20.5)
50 - 59	2674 (31.2)	1344 (31.4)	4018 (31.3)	101 (21.0)	385 (22.3)	486 (22.0)	276 (27.1)	135 (18.4)	411 (23.4)	567 (26.6)	199 (20.4)	766 (24.6)	2984 (27.8)	2979 (29.3)	5963 (28.5)
60 - 69	2068 (24.2)	963 (22.5)	3031 (23.6)	125 (25.9)	473 (27.4)	598 (27.1)	138 (13.5)	66 (9.0)	204 (11.6)	199 (9.3)	117 (12.0)	316 (10.2)	2467 (22.9)	2396 (23.5)	4863 (23.2)
70 - 79	727 (8.5)	385 (9.0)	1112 (8.7)	108 (22.4)	395 (22.9)	503 (22.8)	57 (5.6)	31 (4.2)	88 (5.0)	64 (3.0)	46 (4.7)	110 (3.5)	1316 (12.2)	1461 (14.4)	2777 (13.3)
80 & above	152 (1.8)	82 (1.9)	234 (1.8)	36 (7.5)	157 (9.1)	193 (8.7)	24 (2.4)	14 (1.9)	38 (2.2)	18 (0.8)	19 (1.9)	37 (1.2)	373 (3.5)	560 (5.5)	933 (4.5)
**BMI ≥ 30 kg/m2 (%)**	1840 (21.5)	763 (17.8)	2603 (20.3)	82 (17.0)	417 (24.1)	499 (22.6)	164 (16.1)	141 (19.2)	305 (17.4)	248 (11.6)	170 (17.4)	418 (13.4)	4385 (40.8)	5367 (52.7)	9752 (46.6)
**Smoking (%)**															
Non smoker	1653 (19.3)	986 (23.0)	2639 (20.5)	195 (40.5)	732 (42.4)	927 (41.9)	323 (31.7)	362 (49.3)	685 (39.1)	222 (10.4)	226 (23.1)	448 (14.4)	4002 (37.2)	4874 (47.9)	8876 (42.4)
Current smoker	4487 (52.4)	2316 (54.0)	6803 (53.0)	95 (19.7)	405 (23.4)	500 (22.6)	353 (34.6)	152 (20.7)	505 (28.8)	1453 (68.1)	539 (55.2)	1992 (64.0)	3175 (29.5)	2456 (24.1)	5631 (26.9)
Ex or current smoker	9 (0.1)	5 (0.1)	14 (0.1)	2 (0.4)	2 (0.1)	4 (0.2)	0 (0.0)	1 (0.1)	1 (0.1)	2 (0.1)	0 (0.0)	2 (0.1)	9 (0.1)	8 (0.1)	17 (0.1)
Ex smoker	1973 (23.0)	810 (18.9)	2783 (21.7)	174 (36.1)	526 (30.4)	700 (31.7)	260 (25.5)	176 (24.0)	436 (24.9)	319 (14.9)	162 (16.6)	481 (15.5)	3434 (31.9)	2763 (27.1)	6197 (29.6)
No smoking information	438 (5.1)	168 (3.9)	606 (4.7)	16 (3.3)	63 (3.6)	79 (3.6)	83 (8.1)	43 (5.9)	126 (7.2)	139 (6.5)	50 (5.1)	189 (6.1)	131 (1.2)	76 (0.7)	207 (1.0)
**Comorbidities**															
Barrett's oesophagus (%)	104 (1.2)	30 (0.7)	134 (1.0)	8 (1.7)	12 (0.7)	20 (0.9)	5 (0.5)	1 (0.1)	6 (0.3)	13 (0.6)	3 (0.3)	16 (0.5)	176 (1.6)	98 (1.0)	274 (1.3)
Crohn's disease (%)	29 (0.3)	25 (0.6)	54 (0.4)	23 (4.8)	25 (1.4)	48 (2.2)	5 (0.5)	6 (0.8)	11 (0.6)	7 (0.3)	3 (0.3)	10 (0.3)	107 (1.0)	120 (1.2)	227 (1.1)
Diabetes mellitus (%)	1149 (13.4)	427 (10.0)	1576 (12.3)	82 (17.0)	226 (13.1)	308 (13.9)	127 (12.5)	66 (9.0)	193 (11.0)	183 (8.6)	79 (8.1)	262 (8.4)	2469 (23.0)	2553 (25.1)	5022 (24.0)
Complications of diabetes (%)	270 (3.2)	82 (1.9)	352 (2.7)	29 (6.0)	62 (3.6)	91 (4.1)	23 (2.3)	10 (1.4)	33 (1.9)	49 (2.3)	23 (2.4)	72 (2.3)	691 (6.4)	652 (6.4)	1343 (6.4)
Diverticular disease of intestine (%)	278 (3.2)	152 (3.5)	430 (3.3)	23 (4.8)	123 (7.1)	146 (6.6)	18 (1.8)	14 (1.9)	32 (1.8)	17 (0.8)	21 (2.1)	38 (1.2)	591 (5.5)	848 (8.3)	1439 (6.9)
Dyslipidaemia (%)	940 (11.0)	390 (9.1)	1330 (10.4)	54 (11.2)	218 (12.6)	272 (12.3)	76 (7.5)	37 (5.0)	113 (6.4)	71 (3.3)	36 (3.7)	107 (3.4)	1972 (18.3)	1875 (18.4)	3847 (18.4)
Gastro-oesophageal reflux disease (%)	871 (10.2)	446 (10.4)	1317 (10.3)	56 (11.6)	221 (12.8)	277 (12.5)	77 (7.6)	52 (7.1)	129 (7.4)	162 (7.6)	81 (8.3)	243 (7.8)	1650 (15.3)	2088 (20.5)	3738 (17.9)
Hypertension (%)	2423 (28.3)	1095 (25.6)	3518 (27.4)	128 (26.6)	536 (31.0)	664 (30.0)	168 (16.5)	101 (13.8)	269 (15.3)	231 (10.8)	120 (12.3)	351 (11.3)	3704 (34.5)	3629 (35.7)	7333 (35.0)
Irritable bowel syndrome (%)	289 (3.4)	408 (9.5)	697 (5.4)	27 (5.6)	201 (11.6)	228 (10.3)	45 (4.4)	40 (5.4)	85 (4.8)	59 (2.8)	83 (8.5)	142 (4.6)	745 (6.9)	1775 (17.4)	2520 (12.0)
Jaundice (%)	627 (7.3)	434 (10.1)	1061 (8.3)	80 (16.6)	176 (10.2)	256 (11.6)	72 (7.1)	28 (3.8)	100 (5.7)	77 (3.6)	38 (3.9)	115 (3.7)	257 (2.4)	213 (2.1)	470 (2.2)
Proteinuric kidney diseases (%)	60 (0.7)	18 (0.4)	78 (0.6)	10 (2.1)	15 (0.9)	25 (1.1)	16 (1.6)	10 (1.4)	26 (1.5)	30 (1.4)	10 (1.0)	40 (1.3)	133 (1.2)	117 (1.1)	250 (1.2)
Oesophagitis and oesophageal ulcer (%)	755 (8.8)	340 (7.9)	1095 (8.5)	45 (9.3)	148 (8.6)	193 (8.7)	57 (5.6)	24 (3.3)	81 (4.6)	122 (5.7)	44 (4.5)	166 (5.3)	1103 (10.3)	1233 (12.1)	2336 (11.2)
Proteinuria (%)	2927 (34.2)	1553 (36.2)	4480 (34.9)	213 (44.2)	861 (49.8)	1074 (48.6)	375 (36.8)	296 (40.3)	671 (38.3)	597 (28.0)	383 (39.2)	980 (31.5)	5300 (49.3)	6075 (59.7)	11375 (54.4)
Renal disease (%)	339 (4.0)	218 (5.1)	557 (4.3)	46 (9.5)	194 (11.2)	240 (10.9)	72 (7.1)	45 (6.1)	117 (6.7)	87 (4.1)	62 (6.3)	149 (4.8)	872 (8.1)	1377 (13.5)	2249 (10.7)
**Biomarkers**															
Albumin < 35 g/L (%)	1378 (16.1)	882 (20.6)	2260 (17.6)	75 (15.6)	217 (12.6)	292 (13.2)	62 (6.1)	37 (5.0)	99 (5.6)	142 (6.7)	73 (7.5)	215 (6.9)	438 (4.1)	516 (5.1)	954 (4.6)
Alanine aminotransferase ≥ 35 U/L (%)	1527 (17.8)	650 (15.2)	2177 (16.9)	119 (24.7)	395 (22.9)	514 (23.3)	118 (11.6)	44 (6.0)	162 (9.2)	315 (14.8)	129 (13.2)	444 (14.3)	1605 (14.9)	920 (9.0)	2525 (12.1)
Aspartate transaminase ≥ 40 U/L (%)	570 (6.7)	298 (7.0)	868 (6.8)	39 (8.1)	138 (8.0)	177 (8.0)	41 (4.0)	17 (2.3)	58 (3.3)	85 (4.0)	44 (4.5)	129 (4.1)	327 (3.0)	233 (2.3)	560 (2.7)
Bilirubin ≥ 34.2 µmol/L (%)	1043 (12.2)	657 (15.3)	1700 (13.2)	65 (13.5)	141 (8.2)	206 (9.3)	66 (6.5)	17 (2.3)	83 (4.7)	77 (3.6)	26 (2.7)	103 (3.3)	306 (2.8)	183 (1.8)	489 (2.3)
C reactive protein ≥ 10 mg/L (%)	475 (5.5)	301 (7.0)	776 (6.0)	48 (10.0)	160 (9.3)	208 (9.4)	41 (4.0)	15 (2.0)	56 (3.2)	47 (2.2)	27 (2.8)	74 (2.4)	483 (4.5)	696 (6.8)	1179 (5.6)
Gamma- glutamyltransferase ≥ 48 U/L (%)	1777 (20.8)	852 (19.9)	2629 (20.5)	110 (22.8)	392 (22.7)	502 (22.7)	64 (6.3)	24 (3.3)	88 (5.0)	186 (8.7)	75 (7.7)	261 (8.4)	1157 (10.8)	765 (7.5)	1922 (9.2)
International Normalized Ratio ≥ 1.7 (%)	59 (0.7)	21 (0.5)	80 (0.6)	8 (1.7)	10 (0.6)	18 (0.8)	4 (0.4)	2 (0.3)	6 (0.3)	9 (0.4)	6 (0.6)	15 (0.5)	94 (0.9)	68 (0.7)	162 (0.8)
Platelet ≤ 150 10^9/L (%)	1460 (17.1)	599 (14.0)	2059 (16.0)	55 (11.4)	145 (8.4)	200 (9.0)	89 (8.7)	28 (3.8)	117 (6.7)	222 (10.4)	75 (7.7)	297 (9.5)	618 (5.7)	305 (3.0)	923 (4.4)
Triglycerides ≥ 2 mmol/L (%)	578 (6.8)	200 (4.7)	778 (6.1)	25 (5.2)	67 (3.9)	92 (4.2)	31 (3.0)	11 (1.5)	42 (2.4)	50 (2.3)	15 (1.5)	65 (2.1)	1483 (13.8)	1075 (10.6)	2558 (12.2)
