# Supplementary material for: Increased burden of cardiovascular disease in people with liver disease: unequal geographical variations, risk factors and excess years of life lost
Source: J Transl Med. 2022 Jan 3;20:2. doi: 10.1186/s12967-021-03210-9 (PMC8722174; doi:10.1186/s12967-021-03210-9)
Supplement: Supplementary file 5 — Additional file 5: Age-standardised incidence rates for liver disease. [file 12967_2021_3210_MOESM5_ESM.pdf]

Additional file 5. Age-standardised incidence rates for liver disease.

Liver disease type	Practice region	Incidence rate (per 100,000 person years)	Lower CI	Upper CI
ALD	North East	32.43	24.82	40.05
ALD	North West	34.68	31.75	37.61
ALD	Yorkshire & The Humber	20.78	16.54	25.03
ALD	East Midlands	25.95	20.75	31.16
ALD	West Midlands	26.67	23.72	29.61
ALD	East of England	18.28	15.83	20.74
ALD	South West	24.20	21.57	26.84
ALD	South Central	22.94	20.38	25.49
ALD	London	23.61	21.06	26.16
ALD	South East Coast	22.26	19.86	24.65
ALD	England	24.71	23.76	25.66
Any liver disease	North East	146.04	129.89	162.18
Any liver disease	North West	150.85	144.75	156.96
Any liver disease	Yorkshire & The Humber	86.53	77.86	95.19
Any liver disease	East Midlands	87.59	78.03	97.15
Any liver disease	West Midlands	102.03	96.28	107.79
Any liver disease	East of England	94.61	89.02	100.19
Any liver disease	South West	105.21	99.72	110.71
Any liver disease	South Central	94.10	88.93	99.27
Any liver disease	London	148.58	142.19	154.96
Any liver disease	South East Coast	113.42	108.01	118.82
Any liver disease	England	114.52	112.47	116.56
Autoimmune liver disease	North East	5.87	2.63	9.11
Autoimmune liver disease	North West	4.61	3.54	5.68
Autoimmune liver disease	Yorkshire & The Humber	2.86	1.29	4.44
Autoimmune liver disease	East Midlands	2.90	1.16	4.64
Autoimmune liver disease	West Midlands	3.48	2.42	4.55
Autoimmune liver disease	East of England	3.20	2.17	4.23
Autoimmune liver disease	South West	4.06	2.98	5.14
Autoimmune liver disease	South Central	3.51	2.51	4.51
Autoimmune liver disease	London	3.44	2.47	4.41
Autoimmune liver disease	South East Coast	3.68	2.71	4.66
Autoimmune liver disease	England	3.71	3.34	4.08
HBV	North East	3.07	0.73	5.41
HBV	North West	2.99	2.13	3.86
HBV	Yorkshire & The Humber	0.78	0.00	1.60
HBV	East Midlands	3.14	1.33	4.95
HBV	West Midlands	3.59	2.51	4.67
HBV	East of England	3.27	2.23	4.31
HBV	South West	2.78	1.88	3.67
HBV	South Central	2.47	1.63	3.31
HBV	London	11.40	9.63	13.17
HBV	South East Coast	3.29	2.37	4.21
HBV	England	4.09	3.70	4.47
HCV	North East	9.71	5.54	13.88
HCV	North West	8.96	7.47	10.44
HCV	Yorkshire & The Humber	5.83	3.58	8.08
HCV	East Midlands	6.16	3.62	8.69
HCV	West Midlands	5.29	3.98	6.60
HCV	East of England	4.25	3.06	5.43
HCV	South West	8.15	6.62	9.68
HCV	South Central	4.80	3.63	5.97
HCV	London	5.84	4.57	7.10
HCV	South East Coast	4.73	3.63	5.84
HCV	England	5.98	5.51	6.44
NAFLD	North East	107.62	93.76	121.48
NAFLD	North West	112.16	106.89	117.42
NAFLD	Yorkshire & The Humber	61.09	53.81	68.37
NAFLD	East Midlands	59.09	51.24	66.94
NAFLD	West Midlands	71.43	66.62	76.25
NAFLD	East of England	72.06	67.18	76.93
NAFLD	South West	76.62	71.93	81.31
NAFLD	South Central	68.50	64.08	72.91
NAFLD	London	116.39	110.74	122.04
NAFLD	South East Coast	88.53	83.75	93.30
NAFLD	England	85.48	83.72	87.25
