# Supplementary material for: Increased burden of cardiovascular disease in people with liver disease: unequal geographical variations, risk factors and excess years of life lost
Source: J Transl Med. 2022 Jan 3;20:2. doi: 10.1186/s12967-021-03210-9 (PMC8722174; doi:10.1186/s12967-021-03210-9)
Supplement: Supplementary file 6 — Additional file 6: Age-specific incidence rates for liver disease. [file 12967_2021_3210_MOESM6_ESM.pdf]

Additional file 6. Age-specific incidence rates for liver disease.

Liver disease type	Practice region	Age group	Incidence rate (per 100,000 person years)	Lower CI	Upper CI
ALD	North East	30-39	5.05	0.00	12.86
ALD	North West	30-39	3.57	1.20	5.93
ALD	Yorkshire & The Humber	30-39	2.65	0.00	7.68
ALD	East Midlands	30-39	5.09	0.00	13.32
ALD	West Midlands	30-39	3.00	0.43	5.57
ALD	East of England	30-39	1.88	0.00	4.03
ALD	South West	30-39	3.15	0.55	5.74
ALD	South Central	30-39	2.18	0.15	4.22
ALD	London	30-39	1.34	0.03	2.66
ALD	South East Coast	30-39	2.01	0.19	3.82
ALD	England	30-39	2.50	1.72	3.28
ALD	North East	40-49	6.58	0.00	14.30
ALD	North West	40-49	7.40	4.43	10.38
ALD	Yorkshire & The Humber	40-49	4.66	0.19	9.12
ALD	East Midlands	40-49	4.10	0.00	8.45
ALD	West Midlands	40-49	4.76	2.01	7.50
ALD	East of England	40-49	3.30	1.08	5.51
ALD	South West	40-49	4.95	2.24	7.66
ALD	South Central	40-49	3.62	1.40	5.83
ALD	London	40-49	3.33	1.43	5.24
ALD	South East Coast	40-49	4.45	2.08	6.82
ALD	England	40-49	4.59	3.70	5.47
ALD	North East	50-59	7.24	0.00	14.83
ALD	North West	50-59	9.35	6.14	12.57
ALD	Yorkshire & The Humber	50-59	5.36	0.94	9.78
ALD	East Midlands	50-59	7.61	1.97	13.24
ALD	West Midlands	50-59	7.41	4.07	10.76
ALD	East of England	50-59	5.57	2.72	8.42
ALD	South West	50-59	7.16	4.10	10.22
ALD	South Central	50-59	7.03	4.02	10.03
ALD	London	50-59	7.11	4.12	10.11
ALD	South East Coast	50-59	6.05	3.38	8.73
ALD	England	50-59	7.09	6.01	8.18
ALD	North East	60-69	10.06	0.32	19.81
ALD	North West	60-69	9.11	5.57	12.65
ALD	Yorkshire & The Humber	60-69	5.47	0.69	10.24
ALD	East Midlands	60-69	5.90	0.26	11.54
ALD	West Midlands	60-69	7.86	4.09	11.63
ALD	East of England	60-69	4.55	1.65	7.45
ALD	South West	60-69	5.26	2.41	8.11
ALD	South Central	60-69	5.41	2.48	8.34
ALD	London	60-69	7.22	3.53	10.92
ALD	South East Coast	60-69	6.21	3.19	9.23
ALD	England	60-69	6.56	5.40	7.73
ALD	North East	70-79	2.81	0.00	8.81
ALD	North West	70-79	4.62	1.75	7.48
ALD	Yorkshire & The Humber	70-79	2.11	0.00	5.58
ALD	East Midlands	70-79	2.45	0.00	6.60
ALD	West Midlands	70-79	3.00	0.42	5.57
ALD	East of England	70-79	2.44	0.01	4.88
ALD	South West	70-79	2.86	0.55	5.18
ALD	South Central	70-79	3.72	0.97	6.46
ALD	London	70-79	3.41	0.30	6.52
ALD	South East Coast	70-79	3.08	0.70	5.45
ALD	England	70-79	3.24	2.31	4.17
ALD	North East	80 and above	NA	NA	NA
ALD	North West	80 and above	0.63	0.00	1.94
ALD	Yorkshire & The Humber	80 and above	0.53	0.00	2.61
ALD	East Midlands	80 and above	0.79	0.00	3.63
ALD	West Midlands	80 and above	0.64	0.00	2.05
ALD	East of England	80 and above	0.54	0.00	1.86
ALD	South West	80 and above	0.83	0.00	2.27
ALD	South Central	80 and above	0.98	0.00	2.66
ALD	London	80 and above	1.19	0.00	3.31
ALD	South East Coast	80 and above	0.46	0.00	1.52
ALD	England	80 and above	0.73	0.21	1.25
Any liver disease	North East	30-39	20.21	4.59	35.82
Any liver disease	North West	30-39	17.99	12.67	23.31
Any liver disease	Yorkshire & The Humber	30-39	13.60	2.23	24.98
Any liver disease	East Midlands	30-39	10.66	0.00	22.56
Any liver disease	West Midlands	30-39	13.08	7.71	18.45
Any liver disease	East of England	30-39	10.44	5.38	15.50
Any liver disease	South West	30-39	14.98	9.31	20.65
Any liver disease	South Central	30-39	11.65	6.95	16.35
Any liver disease	London	30-39	14.64	10.30	18.97
Any liver disease	South East Coast	30-39	11.92	7.50	16.35
Any liver disease	England	30-39	13.84	12.01	15.67
Any liver disease	North East	40-49	24.53	9.61	39.44
Any liver disease	North West	40-49	26.43	20.81	32.05
Any liver disease	Yorkshire & The Humber	40-49	12.84	5.43	20.26
Any liver disease	East Midlands	40-49	15.50	7.05	23.96
Any liver disease	West Midlands	40-49	17.56	12.28	22.83
Any liver disease	East of England	40-49	15.49	10.69	20.30
Any liver disease	South West	40-49	18.62	13.36	23.88
Any liver disease	South Central	40-49	14.86	10.38	19.35
Any liver disease	London	40-49	20.65	15.91	25.38
Any liver disease	South East Coast	40-49	19.43	14.48	24.38
Any liver disease	England	40-49	18.99	17.18	20.79
Any liver disease	North East	50-59	37.06	19.90	54.22
Any liver disease	North West	50-59	41.28	34.52	48.04
Any liver disease	Yorkshire & The Humber	50-59	21.20	12.41	29.99
Any liver disease	East Midlands	50-59	23.12	13.30	32.95
Any liver disease	West Midlands	50-59	24.67	18.57	30.78
Any liver disease	East of England	50-59	24.79	18.78	30.80
Any liver disease	South West	50-59	27.26	21.29	33.23
Any liver disease	South Central	50-59	23.61	18.10	29.12
Any liver disease	London	50-59	38.13	31.20	45.06
Any liver disease	South East Coast	50-59	29.15	23.28	35.03
Any liver disease	England	50-59	29.77	27.55	31.98
Any liver disease	North East	60-69	32.26	14.81	49.72
Any liver disease	North West	60-69	36.52	29.42	43.61
Any liver disease	Yorkshire & The Humber	60-69	21.08	11.70	30.45
Any liver disease	East Midlands	60-69	19.63	9.33	29.92
Any liver disease	West Midlands	60-69	25.87	19.03	32.71
Any liver disease	East of England	60-69	22.20	15.79	28.61
Any liver disease	South West	60-69	22.05	16.23	27.88
Any liver disease	South Central	60-69	21.70	15.84	27.56
Any liver disease	London	60-69	37.37	28.97	45.77
Any liver disease	South East Coast	60-69	27.62	21.25	33.98
Any liver disease	England	60-69	27.17	24.80	29.54
Any liver disease	North East	70-79	22.73	5.64	39.82
Any liver disease	North West	70-79	21.85	15.62	28.08
Any liver disease	Yorkshire & The Humber	70-79	12.92	4.35	21.49
Any liver disease	East Midlands	70-79	12.72	3.27	22.17
Any liver disease	West Midlands	70-79	15.22	9.42	21.02
Any liver disease	East of England	70-79	16.26	9.97	22.54
Any liver disease	South West	70-79	16.87	11.24	22.49
Any liver disease	South Central	70-79	15.98	10.29	21.67
Any liver disease	London	70-79	26.83	18.10	35.56
Any liver disease	South East Coast	70-79	18.84	12.96	24.71
Any liver disease	England	70-79	18.23	16.03	20.44
Any liver disease	North East	80 and above	9.26	0.00	22.03
Any liver disease	North West	80 and above	6.79	2.52	11.05
Any liver disease	Yorkshire & The Humber	80 and above	4.89	0.00	11.18
Any liver disease	East Midlands	80 and above	5.96	0.00	13.71
Any liver disease	West Midlands	80 and above	5.63	1.48	9.79
Any liver disease	East of England	80 and above	5.43	1.27	9.58
Any liver disease	South West	80 and above	5.43	1.73	9.14
Any liver disease	South Central	80 and above	6.30	2.04	10.55
Any liver disease	London	80 and above	10.96	4.52	17.39
Any liver disease	South East Coast	80 and above	6.46	2.45	10.46
Any liver disease	England	80 and above	6.52	4.97	8.07
Autoimmune liver disease	North East	30-39	NA	NA	NA
Autoimmune liver disease	North West	30-39	0.38	0.00	1.16
Autoimmune liver disease	Yorkshire & The Humber	30-39	NA	NA	NA
Autoimmune liver disease	East Midlands	30-39	NA	NA	NA
Autoimmune liver disease	West Midlands	30-39	NA	NA	NA
Autoimmune liver disease	East of England	30-39	NA	NA	NA
Autoimmune liver disease	South West	30-39	0.37	0.00	1.27
Autoimmune liver disease	South Central	30-39	0.46	0.00	1.40
Autoimmune liver disease	London	30-39	NA	NA	NA
Autoimmune liver disease	South East Coast	30-39	0.34	0.00	1.10
Autoimmune liver disease	England	30-39	0.30	0.03	0.57
Autoimmune liver disease	North East	40-49	NA	NA	NA
Autoimmune liver disease	North West	40-49	0.39	0.00	1.08
Autoimmune liver disease	Yorkshire & The Humber	40-49	NA	NA	NA
Autoimmune liver disease	East Midlands	40-49	NA	NA	NA
Autoimmune liver disease	West Midlands	40-49	0.42	0.00	1.23
Autoimmune liver disease	East of England	40-49	NA	NA	NA
Autoimmune liver disease	South West	40-49	0.44	0.00	1.25
Autoimmune liver disease	South Central	40-49	0.45	0.00	1.22
Autoimmune liver disease	London	40-49	0.54	0.00	1.30
Autoimmune liver disease	South East Coast	40-49	NA	NA	NA
Autoimmune liver disease	England	40-49	0.35	0.11	0.60
Autoimmune liver disease	North East	50-59	NA	NA	NA
Autoimmune liver disease	North West	50-59	1.01	0.00	2.07
Autoimmune liver disease	Yorkshire & The Humber	50-59	0.89	0.00	2.70
Autoimmune liver disease	East Midlands	50-59	0.88	0.00	2.79
Autoimmune liver disease	West Midlands	50-59	0.69	0.00	1.71
Autoimmune liver disease	East of England	50-59	0.61	0.00	1.56
Autoimmune liver disease	South West	50-59	0.73	0.00	1.71
Autoimmune liver disease	South Central	50-59	0.45	0.00	1.21
Autoimmune liver disease	London	50-59	0.53	0.00	1.35
Autoimmune liver disease	South East Coast	50-59	0.66	0.00	1.55
Autoimmune liver disease	England	50-59	0.70	0.36	1.04
Autoimmune liver disease	North East	60-69	2.06	0.00	6.48
Autoimmune liver disease	North West	60-69	1.28	0.00	2.61
Autoimmune liver disease	Yorkshire & The Humber	60-69	0.68	0.00	2.37
Autoimmune liver disease	East Midlands	60-69	NA	NA	NA
Autoimmune liver disease	West Midlands	60-69	1.04	0.00	2.41
Autoimmune liver disease	East of England	60-69	0.51	0.00	1.47
Autoimmune liver disease	South West	60-69	1.05	0.00	2.32
Autoimmune liver disease	South Central	60-69	0.91	0.00	2.11
Autoimmune liver disease	London	60-69	0.77	0.00	1.98
Autoimmune liver disease	South East Coast	60-69	1.36	0.00	2.78
Autoimmune liver disease	England	60-69	1.00	0.54	1.45
Autoimmune liver disease	North East	70-79	1.40	0.00	5.65
Autoimmune liver disease	North West	70-79	1.13	0.00	2.54
Autoimmune liver disease	Yorkshire & The Humber	70-79	NA	NA	NA
Autoimmune liver disease	East Midlands	70-79	0.77	0.00	3.09
Autoimmune liver disease	West Midlands	70-79	0.82	0.00	2.17
Autoimmune liver disease	East of England	70-79	1.17	0.00	2.85
Autoimmune liver disease	South West	70-79	1.23	0.00	2.75
Autoimmune liver disease	South Central	70-79	0.75	0.00	1.99
Autoimmune liver disease	London	70-79	0.87	0.00	2.44
Autoimmune liver disease	South East Coast	70-79	0.84	0.00	2.08
Autoimmune liver disease	England	70-79	0.95	0.45	1.45
Autoimmune liver disease	North East	80 and above	NA	NA	NA
Autoimmune liver disease	North West	80 and above	0.42	0.00	1.49
Autoimmune liver disease	Yorkshire & The Humber	80 and above	NA	NA	NA
Autoimmune liver disease	East Midlands	80 and above	0.66	0.00	3.25
Autoimmune liver disease	West Midlands	80 and above	0.36	0.00	1.42
Autoimmune liver disease	East of England	80 and above	0.38	0.00	1.47
Autoimmune liver disease	South West	80 and above	0.23	0.00	1.00
Autoimmune liver disease	South Central	80 and above	0.49	0.00	1.68
Autoimmune liver disease	London	80 and above	0.60	0.00	2.09
Autoimmune liver disease	South East Coast	80 and above	0.39	0.00	1.38
Autoimmune liver disease	England	80 and above	0.40	0.02	0.79
HBV	North East	30-39	NA	NA	NA
HBV	North West	30-39	0.71	0.00	1.77
HBV	Yorkshire & The Humber	30-39	NA	NA	NA
HBV	East Midlands	30-39	NA	NA	NA
HBV	West Midlands	30-39	1.38	0.00	3.13
HBV	East of England	30-39	1.11	0.00	2.76
HBV	South West	30-39	1.12	0.00	2.68
HBV	South Central	30-39	0.93	0.00	2.25
HBV	London	30-39	2.42	0.66	4.18
HBV	South East Coast	30-39	0.75	0.00	1.85
HBV	England	30-39	1.21	0.67	1.75
HBV	North East	40-49	NA	NA	NA
HBV	North West	40-49	0.83	0.00	1.82
HBV	Yorkshire & The Humber	40-49	NA	NA	NA
HBV	East Midlands	40-49	NA	NA	NA
HBV	West Midlands	40-49	0.52	0.00	1.43
HBV	East of England	40-49	0.59	0.00	1.53
HBV	South West	40-49	0.34	0.00	1.06
HBV	South Central	40-49	0.45	0.00	1.22
HBV	London	40-49	2.08	0.58	3.58
HBV	South East Coast	40-49	0.79	0.00	1.79
HBV	England	40-49	0.82	0.44	1.19
HBV	North East	50-59	NA	NA	NA
HBV	North West	50-59	0.74	0.00	1.64
HBV	Yorkshire & The Humber	50-59	NA	NA	NA
HBV	East Midlands	50-59	NA	NA	NA
HBV	West Midlands	50-59	0.85	0.00	1.98
HBV	East of England	50-59	0.77	0.00	1.82
HBV	South West	50-59	0.50	0.00	1.32
HBV	South Central	50-59	0.68	0.00	1.61
HBV	London	50-59	2.78	0.91	4.66
HBV	South East Coast	50-59	0.66	0.00	1.55
HBV	England	50-59	0.94	0.55	1.33
HBV	North East	60-69	NA	NA	NA
HBV	North West	60-69	0.30	0.00	0.94
HBV	Yorkshire & The Humber	60-69	NA	NA	NA
HBV	East Midlands	60-69	NA	NA	NA
HBV	West Midlands	60-69	0.35	0.00	1.14
HBV	East of England	60-69	0.25	0.00	0.94
HBV	South West	60-69	0.42	0.00	1.23
HBV	South Central	60-69	NA	NA	NA
HBV	London	60-69	2.12	0.12	4.11
HBV	South East Coast	60-69	0.64	0.00	1.61
HBV	England	60-69	0.54	0.20	0.87
HBV	North East	70-79	NA	NA	NA
HBV	North West	70-79	0.31	0.00	1.05
HBV	Yorkshire & The Humber	70-79	NA	NA	NA
HBV	East Midlands	70-79	0.77	0.00	3.09
HBV	West Midlands	70-79	0.29	0.00	1.09
HBV	East of England	70-79	0.42	0.00	1.44
HBV	South West	70-79	0.29	0.00	1.02
HBV	South Central	70-79	NA	NA	NA
HBV	London	70-79	1.36	0.00	3.33
HBV	South East Coast	70-79	0.32	0.00	1.09
HBV	England	70-79	0.40	0.07	0.73
HBV	North East	80 and above	NA	NA	NA
HBV	North West	80 and above	NA	NA	NA
HBV	Yorkshire & The Humber	80 and above	NA	NA	NA
HBV	East Midlands	80 and above	NA	NA	NA
HBV	West Midlands	80 and above	0.20	0.00	0.99
HBV	East of England	80 and above	NA	NA	NA
HBV	South West	80 and above	NA	NA	NA
HBV	South Central	80 and above	NA	NA	NA
HBV	London	80 and above	0.64	0.00	2.21
HBV	South East Coast	80 and above	NA	NA	NA
HBV	England	80 and above	0.18	0.00	0.44
HCV	North East	30-39	5.05	0.00	12.86
HCV	North West	30-39	2.03	0.24	3.82
HCV	Yorkshire & The Humber	30-39	2.99	0.00	8.31
HCV	East Midlands	30-39	NA	NA	NA
HCV	West Midlands	30-39	1.69	0.00	3.62
HCV	East of England	30-39	0.60	0.00	1.81
HCV	South West	30-39	3.37	0.68	6.06
HCV	South Central	30-39	1.39	0.00	3.01
HCV	London	30-39	0.76	0.00	1.75
HCV	South East Coast	30-39	0.75	0.00	1.85
HCV	England	30-39	1.58	0.96	2.19
HCV	North East	40-49	2.99	0.00	8.20
HCV	North West	40-49	2.91	1.05	4.78
HCV	Yorkshire & The Humber	40-49	1.41	0.00	3.87
HCV	East Midlands	40-49	1.37	0.00	3.88
HCV	West Midlands	40-49	1.41	0.00	2.91
HCV	East of England	40-49	1.18	0.00	2.51
HCV	South West	40-49	2.25	0.42	4.08
HCV	South Central	40-49	1.29	0.00	2.62
HCV	London	40-49	0.65	0.00	1.48
HCV	South East Coast	40-49	1.41	0.08	2.75
HCV	England	40-49	1.59	1.07	2.11
HCV	North East	50-59	1.67	0.00	5.31
HCV	North West	50-59	2.83	1.06	4.60
HCV	Yorkshire & The Humber	50-59	NA	NA	NA
HCV	East Midlands	50-59	1.90	0.00	4.72
HCV	West Midlands	50-59	1.38	0.00	2.82
HCV	East of England	50-59	1.33	0.00	2.72
HCV	South West	50-59	1.24	0.00	2.51
HCV	South Central	50-59	0.95	0.00	2.05
HCV	London	50-59	1.81	0.30	3.32
HCV	South East Coast	50-59	1.16	0.00	2.33
HCV	England	50-59	1.53	1.03	2.03
HCV	North East	60-69	NA	NA	NA
HCV	North West	60-69	0.56	0.00	1.45
HCV	Yorkshire & The Humber	60-69	0.80	0.00	2.62
HCV	East Midlands	60-69	1.03	0.00	3.39
HCV	West Midlands	60-69	0.40	0.00	1.24
HCV	East of England	60-69	0.76	0.00	1.94
HCV	South West	60-69	0.93	0.00	2.12
HCV	South Central	60-69	0.65	0.00	1.66
HCV	London	60-69	1.39	0.00	3.01
HCV	South East Coast	60-69	1.08	0.00	2.34
HCV	England	60-69	0.81	0.40	1.22
HCV	North East	70-79	NA	NA	NA
HCV	North West	70-79	0.54	0.00	1.53
HCV	Yorkshire & The Humber	70-79	NA	NA	NA
HCV	East Midlands	70-79	NA	NA	NA
HCV	West Midlands	70-79	0.29	0.00	1.09
HCV	East of England	70-79	NA	NA	NA
HCV	South West	70-79	0.33	0.00	1.11
HCV	South Central	70-79	0.44	0.00	1.39
HCV	London	70-79	1.18	0.00	3.00
HCV	South East Coast	70-79	0.20	0.00	0.81
HCV	England	70-79	0.39	0.07	0.71
HCV	North East	80 and above	NA	NA	NA
HCV	North West	80 and above	NA	NA	NA
HCV	Yorkshire & The Humber	80 and above	NA	NA	NA
HCV	East Midlands	80 and above	NA	NA	NA
HCV	West Midlands	80 and above	NA	NA	NA
HCV	East of England	80 and above	NA	NA	NA
HCV	South West	80 and above	NA	NA	NA
HCV	South Central	80 and above	NA	NA	NA
HCV	London	80 and above	NA	NA	NA
HCV	South East Coast	80 and above	NA	NA	NA
HCV	England	80 and above	0.08	0.00	0.26
NAFLD	North East	30-39	13.05	0.50	25.60
NAFLD	North West	30-39	12.84	8.34	17.33
NAFLD	Yorkshire & The Humber	30-39	7.30	0.00	15.63
NAFLD	East Midlands	30-39	6.02	0.00	14.97
NAFLD	West Midlands	30-39	7.85	3.69	12.01
NAFLD	East of England	30-39	7.36	3.11	11.61
NAFLD	South West	30-39	9.29	4.82	13.75
NAFLD	South Central	30-39	7.68	3.86	11.50
NAFLD	London	30-39	10.92	7.18	14.67
NAFLD	South East Coast	30-39	9.11	5.24	12.98
NAFLD	England	30-39	9.53	8.01	11.04
NAFLD	North East	40-49	17.05	4.61	29.48
NAFLD	North West	40-49	17.92	13.30	22.55
NAFLD	Yorkshire & The Humber	40-49	7.62	1.91	13.33
NAFLD	East Midlands	40-49	11.09	3.94	18.25
NAFLD	West Midlands	40-49	12.23	7.83	16.63
NAFLD	East of England	40-49	11.61	7.45	15.77
NAFLD	South West	40-49	12.74	8.39	17.09
NAFLD	South Central	40-49	10.40	6.65	14.15
NAFLD	London	40-49	15.66	11.54	19.79
NAFLD	South East Coast	40-49	14.52	10.24	18.80
NAFLD	England	40-49	13.52	12.00	15.04
NAFLD	North East	50-59	28.41	13.39	43.44
NAFLD	North West	50-59	30.75	24.91	36.58
NAFLD	Yorkshire & The Humber	50-59	15.70	8.14	23.27
NAFLD	East Midlands	50-59	14.63	6.82	22.45
NAFLD	West Midlands	50-59	17.36	12.24	22.49
NAFLD	East of England	50-59	18.50	13.31	23.69
NAFLD	South West	50-59	20.42	15.25	25.59
NAFLD	South Central	50-59	17.21	12.51	21.91
NAFLD	London	50-59	29.16	23.10	35.22
NAFLD	South East Coast	50-59	23.22	17.98	28.46
NAFLD	England	50-59	22.25	20.34	24.16
NAFLD	North East	60-69	21.93	7.54	36.32
NAFLD	North West	60-69	28.42	22.16	34.67
NAFLD	Yorkshire & The Humber	60-69	15.04	7.12	22.95
NAFLD	East Midlands	60-69	12.98	4.61	21.36
NAFLD	West Midlands	60-69	18.20	12.46	23.94
NAFLD	East of England	60-69	17.54	11.85	23.24
NAFLD	South West	60-69	16.54	11.49	21.59
NAFLD	South Central	60-69	16.41	11.31	21.51
NAFLD	London	60-69	30.09	22.55	37.62
NAFLD	South East Coast	60-69	20.80	15.28	26.32
NAFLD	England	60-69	20.59	18.53	22.66
NAFLD	North East	70-79	19.07	3.42	34.73
NAFLD	North West	70-79	16.65	11.21	22.08
NAFLD	Yorkshire & The Humber	70-79	11.18	3.20	19.16
NAFLD	East Midlands	70-79	10.12	1.69	18.54
NAFLD	West Midlands	70-79	11.21	6.23	16.19
NAFLD	East of England	70-79	12.75	7.18	18.32
NAFLD	South West	70-79	13.26	8.28	18.25
NAFLD	South Central	70-79	12.08	7.13	17.03
NAFLD	London	70-79	21.69	13.84	29.53
NAFLD	South East Coast	70-79	15.40	10.09	20.71
NAFLD	England	70-79	14.32	12.36	16.27
NAFLD	North East	80 and above	8.10	0.00	20.05
NAFLD	North West	80 and above	5.59	1.72	9.46
NAFLD	Yorkshire & The Humber	80 and above	4.25	0.00	10.12
NAFLD	East Midlands	80 and above	4.24	0.00	10.78
NAFLD	West Midlands	80 and above	4.59	0.84	8.34
NAFLD	East of England	80 and above	4.30	0.60	8.00
NAFLD	South West	80 and above	4.37	1.05	7.69
NAFLD	South Central	80 and above	4.71	1.03	8.39
NAFLD	London	80 and above	8.88	3.08	14.67
NAFLD	South East Coast	80 and above	5.48	1.79	9.17
NAFLD	England	80 and above	5.27	3.88	6.67
