# Supplementary material for: Increased burden of cardiovascular disease in people with liver disease: unequal geographical variations, risk factors and excess years of life lost
Source: J Transl Med. 2022 Jan 3;20:2. doi: 10.1186/s12967-021-03210-9 (PMC8722174; doi:10.1186/s12967-021-03210-9)
Supplement: Supplementary file 7 — Additional file 7: Age-standardised incidence rates for cardiovascular disease in patients with liver disease. [file 12967_2021_3210_MOESM7_ESM.pdf]

Additional file 7. Age-standardised incidence rates for cardiovascular disease in patients with liver

Liver disease type	Practice region	Incidence rate (per 100,000 person years)	Lower CI	Upper CI
**Any liver disease**	North East	3,179.00	2,422.97	3,935.03
**Any liver disease**	North West	2,960.34	2,703.90	3,216.78
**Any liver disease**	Yorkshire & The Humber	2,559.82	1,987.19	3,132.45
**Any liver disease**	East Midlands	2,856.19	2,202.45	3,509.92
**Any liver disease**	West Midlands	2,548.04	2,194.39	2,901.70
**Any liver disease**	East of England	2,465.08	2,112.92	2,817.24
**Any liver disease**	South West	2,626.68	2,322.48	2,930.87
**Any liver disease**	South Central	2,571.68	2,238.61	2,904.75
**Any liver disease**	London	2,587.40	2,321.93	2,852.86
**Any liver disease**	South East Coast	2,420.22	2,098.16	2,742.28
**Any liver disease**	England	2,634.56	2,524.35	2,744.78
**ALD**	North East	3,826.94	2,431.06	5,222.82
**ALD**	North West	3,677.71	3,184.45	4,170.97
**ALD**	Yorkshire & The Humber	2,804.66	1,767.17	3,842.14
**ALD**	East Midlands	3,116.80	1,952.88	4,280.71
**ALD**	West Midlands	3,129.29	2,497.71	3,760.87
**ALD**	East of England	3,022.21	2,285.28	3,759.13
**ALD**	South West	2,765.31	2,191.18	3,339.44
**ALD**	South Central	2,945.78	2,280.22	3,611.35
**ALD**	London	3,339.78	2,740.14	3,939.43
**ALD**	South East Coast	3,025.06	2,371.32	3,678.79
**ALD**	England	3,173.27	2,955.29	3,391.26
**Autoimmune liver disease**	North East	3,512.42	652.94	6,371.89
**Autoimmune liver disease**	North West	2,817.86	1,834.20	3,801.51
**Autoimmune liver disease**	Yorkshire & The Humber	1,329.06	77.78	2,580.34
**Autoimmune liver disease**	East Midlands	1,776.37	63.53	3,489.20
**Autoimmune liver disease**	West Midlands	1,249.10	405.76	2,092.45
**Autoimmune liver disease**	East of England	2,504.67	1,282.96	3,726.39
**Autoimmune liver disease**	South West	2,080.02	1,078.49	3,081.55
**Autoimmune liver disease**	South Central	1,817.02	817.93	2,816.10
**Autoimmune liver disease**	London	2,216.58	1,231.04	3,202.12
**Autoimmune liver disease**	South East Coast	2,078.04	1,134.48	3,021.60
**Autoimmune liver disease**	England	2,084.81	1,731.82	2,437.81
**HBV**	North East	NA	NA	NA
**HBV**	North West	1,670.97	1,117.50	2,224.44
**HBV**	Yorkshire & The Humber	NA	NA	NA
**HBV**	East Midlands	2,015.42	295.72	3,735.13
**HBV**	West Midlands	3,119.52	1,925.81	4,313.23
**HBV**	East of England	1,182.07	494.74	1,869.39
**HBV**	South West	1,254.62	655.59	1,853.65
**HBV**	South Central	1,483.32	780.74	2,185.91
**HBV**	London	1,668.56	1,164.91	2,172.20
**HBV**	South East Coast	1,378.24	610.86	2,145.61
**HBV**	England	1,550.04	1,312.60	1,787.48
**HCV**	North East	2,634.96	701.28	4,568.64
**HCV**	North West	2,811.84	2,249.37	3,374.31
**HCV**	Yorkshire & The Humber	1,543.42	387.24	2,699.60
**HCV**	East Midlands	525.88	0.00	1,184.04
**HCV**	West Midlands	3,674.50	2,442.75	4,906.25
**HCV**	East of England	1,906.32	1,193.89	2,618.76
**HCV**	South West	1,685.73	1,121.44	2,250.02
**HCV**	South Central	3,713.75	2,706.46	4,721.05
**HCV**	London	1,645.29	1,166.84	2,123.74
**HCV**	South East Coast	2,826.50	1,791.92	3,861.09
**HCV**	England	1,999.59	1,766.44	2,232.74
**NAFLD**	North East	2,813.15	1,797.29	3,829.01
**NAFLD**	North West	3,156.57	2,758.49	3,554.65
**NAFLD**	Yorkshire & The Humber	2,950.51	2,016.78	3,884.23
**NAFLD**	East Midlands	3,325.11	2,268.82	4,381.41
**NAFLD**	West Midlands	2,771.89	2,204.98	3,338.80
**NAFLD**	East of England	2,834.76	2,278.13	3,391.40
**NAFLD**	South West	3,036.92	2,551.34	3,522.50
**NAFLD**	South Central	2,746.64	2,242.82	3,250.47
**NAFLD**	London	2,763.51	2,363.60	3,163.41
**NAFLD**	South East Coast	2,674.99	2,201.27	3,148.72
**NAFLD**	England	2,878.02	2,708.09	3,047.96
