# Supplementary material for: Increased burden of cardiovascular disease in people with liver disease: unequal geographical variations, risk factors and excess years of life lost
Source: J Transl Med. 2022 Jan 3;20:2. doi: 10.1186/s12967-021-03210-9 (PMC8722174; doi:10.1186/s12967-021-03210-9)
Supplement: Supplementary file 8 — Additional file 8: Age-standardised incidence rates for cardiovascular disease in patients without liver disease. [file 12967_2021_3210_MOESM8_ESM.pdf]

Additional file 8. Age-standardised incidence rates for cardiovascular disease in patients without liver disease.

Practice region	Incidence rate (per 100,000 person years)	Lower CI	Upper CI
**North East**	1,454.62	1,400.15	1,509.09
**North West**	1,536.53	1,515.84	1,557.23
**Yorkshire & The Humber**	1,302.05	1,266.20	1,337.91
**East Midlands**	1,338.47	1,298.67	1,378.28
**West Midlands**	1,361.91	1,339.73	1,384.09
**East of England**	1,246.88	1,225.54	1,268.22
**South West**	1,333.81	1,313.06	1,354.55
**South Central**	1,258.65	1,238.91	1,278.38
**London**	1,290.80	1,271.45	1,310.14
**South East Coast**	1,321.27	1,302.10	1,340.44
**England**	1,339.65	1,332.30	1,346.99
