# Supplementary material for: Increased burden of cardiovascular disease in people with liver disease: unequal geographical variations, risk factors and excess years of life lost
Source: J Transl Med. 2022 Jan 3;20:2. doi: 10.1186/s12967-021-03210-9 (PMC8722174; doi:10.1186/s12967-021-03210-9)
Supplement: Supplementary file 9 — Additional file 9: Age-specific incidence rates for cardiovascular disease in patients with liver disease. [file 12967_2021_3210_MOESM9_ESM.pdf]

Additional file 9. Age-specific incidence rates for cardiovascular disease in patients with liver disease.

Liver disease type	Practice region	Age group	Incidence rate (per 100,000 person years)	Lower CI	Upper CI
**Any liver disease**	North East	40-49	278.77	0.00	789.78
**Any liver disease**	North East	60-69	508.66	0.00	1,110.59
**Any liver disease**	North East	50-59	607.41	0.00	1,241.79
**Any liver disease**	North East	70-79	801.53	0.00	1,861.20
**Any liver disease**	North East	80 and above	910.68	0.00	2,889.22
**Any liver disease**	North East	30-39	NA	NA	NA
**Any liver disease**	North West	30-39	97.60	0.00	248.40
**Any liver disease**	North West	40-49	168.13	41.06	295.20
**Any liver disease**	North West	80 and above	776.84	101.28	1,452.41
**Any liver disease**	North West	50-59	381.82	211.59	552.04
**Any liver disease**	North West	60-69	591.49	337.95	845.02
**Any liver disease**	North West	70-79	944.46	513.56	1,375.37
**Any liver disease**	Yorkshire & The Humber	40-49	198.24	0.00	583.82
**Any liver disease**	Yorkshire & The Humber	50-59	392.15	0.00	802.15
**Any liver disease**	Yorkshire & The Humber	60-69	435.44	0.00	886.20
**Any liver disease**	Yorkshire & The Humber	80 and above	576.85	0.00	1,918.90
**Any liver disease**	Yorkshire & The Humber	70-79	713.24	0.00	1,526.32
**Any liver disease**	Yorkshire & The Humber	30-39	NA	NA	NA
**Any liver disease**	East Midlands	40-49	252.54	0.00	671.31
**Any liver disease**	East Midlands	50-59	354.60	0.00	777.38
**Any liver disease**	East Midlands	60-69	534.22	0.00	1,113.09
**Any liver disease**	East Midlands	80 and above	792.30	0.00	2,571.15
**Any liver disease**	East Midlands	70-79	922.54	0.00	1,944.32
**Any liver disease**	East Midlands	30-39	NA	NA	NA
**Any liver disease**	West Midlands	30-39	126.96	0.00	404.32
**Any liver disease**	West Midlands	40-49	194.89	0.00	418.45
**Any liver disease**	West Midlands	80 and above	633.12	0.00	1,483.30
**Any liver disease**	West Midlands	50-59	393.49	124.90	662.07
**Any liver disease**	West Midlands	60-69	446.15	150.57	741.74
**Any liver disease**	West Midlands	70-79	753.43	247.93	1,258.93
**Any liver disease**	East of England	40-49	180.75	0.00	392.77
**Any liver disease**	East of England	80 and above	723.66	0.00	1,536.32
**Any liver disease**	East of England	50-59	308.19	78.03	538.35
**Any liver disease**	East of England	70-79	679.56	188.12	1,171.00
**Any liver disease**	East of England	60-69	544.43	196.27	892.58
**Any liver disease**	East of England	30-39	NA	NA	NA
**Any liver disease**	South West	40-49	113.71	0.00	257.29
**Any liver disease**	South West	30-39	157.63	0.00	424.32
**Any liver disease**	South West	80 and above	861.57	24.25	1,698.89
**Any liver disease**	South West	60-69	432.93	184.89	680.97
**Any liver disease**	South West	50-59	417.33	187.89	646.78
**Any liver disease**	South West	70-79	643.51	233.28	1,053.73
**Any liver disease**	South Central	40-49	194.57	0.00	408.65
**Any liver disease**	South Central	80 and above	792.03	0.00	1,666.70
**Any liver disease**	South Central	50-59	313.39	93.93	532.85
**Any liver disease**	South Central	70-79	616.67	196.35	1,036.99
**Any liver disease**	South Central	60-69	598.07	270.15	925.99
**Any liver disease**	South Central	30-39	NA	NA	NA
**Any liver disease**	London	80 and above	653.11	0.00	1,332.38
**Any liver disease**	London	40-49	156.77	14.95	298.59
**Any liver disease**	London	50-59	329.30	157.92	500.68
**Any liver disease**	London	60-69	502.81	255.08	750.54
**Any liver disease**	London	70-79	905.12	418.42	1,391.82
**Any liver disease**	London	30-39	NA	NA	NA
**Any liver disease**	South East Coast	40-49	139.73	0.00	320.97
**Any liver disease**	South East Coast	80 and above	644.45	0.00	1,345.46
**Any liver disease**	South East Coast	50-59	356.39	113.08	599.70
**Any liver disease**	South East Coast	60-69	529.38	223.46	835.30
**Any liver disease**	South East Coast	70-79	694.77	263.38	1,126.16
**Any liver disease**	South East Coast	30-39	NA	NA	NA
**Any liver disease**	England	30-39	84.05	15.45	152.66
**Any liver disease**	England	40-49	168.97	106.99	230.95
**Any liver disease**	England	50-59	366.30	289.40	443.20
**Any liver disease**	England	60-69	518.97	417.48	620.45
**Any liver disease**	England	80 and above	730.07	452.39	1,007.75
**Any liver disease**	England	70-79	766.21	601.94	930.48
**ALD**	North East	60-69	423.48	0.00	1,275.60
**ALD**	North East	70-79	637.82	0.00	2,159.21
**ALD**	North East	50-59	697.53	0.00	1,817.02
**ALD**	North East	80 and above	1,375.38	0.00	6,243.54
**ALD**	North East	30-39	NA	NA	NA
**ALD**	North East	40-49	NA	NA	NA
**ALD**	North West	30-39	184.74	0.00	626.73
**ALD**	North West	40-49	204.12	0.00	455.20
**ALD**	North West	80 and above	874.38	0.00	2,459.11
**ALD**	North West	50-59	479.26	164.90	793.62
**ALD**	North West	60-69	749.10	290.71	1,207.48
**ALD**	North West	70-79	1,186.11	349.56	2,022.66
**ALD**	Yorkshire & The Humber	50-59	258.03	0.00	820.00
**ALD**	Yorkshire & The Humber	60-69	598.72	0.00	1,427.15
**ALD**	Yorkshire & The Humber	70-79	746.40	0.00	2,337.98
**ALD**	Yorkshire & The Humber	80 and above	1,022.91	0.00	4,380.92
**ALD**	Yorkshire & The Humber	30-39	NA	NA	NA
**ALD**	Yorkshire & The Humber	40-49	NA	NA	NA
**ALD**	East Midlands	50-59	350.35	0.00	1,056.45
**ALD**	East Midlands	60-69	671.45	0.00	1,653.27
**ALD**	East Midlands	70-79	1,061.21	0.00	3,042.72
**ALD**	East Midlands	30-39	NA	NA	NA
**ALD**	East Midlands	40-49	NA	NA	NA
**ALD**	East Midlands	80 and above	NA	NA	NA
**ALD**	West Midlands	40-49	337.21	0.00	832.66
**ALD**	West Midlands	80 and above	728.30	0.00	2,421.41
**ALD**	West Midlands	70-79	877.65	0.00	1,769.24
**ALD**	West Midlands	60-69	518.33	29.91	1,006.75
**ALD**	West Midlands	50-59	503.23	36.48	969.98
**ALD**	West Midlands	30-39	NA	NA	NA
**ALD**	East of England	40-49	263.29	0.00	775.24
**ALD**	East of England	50-59	392.60	0.00	860.61
**ALD**	East of England	60-69	539.35	0.00	1,165.57
**ALD**	East of England	80 and above	737.53	0.00	2,875.85
**ALD**	East of England	70-79	903.78	0.00	1,904.86
**ALD**	East of England	30-39	NA	NA	NA
**ALD**	South West	40-49	117.92	0.00	408.17
**ALD**	South West	80 and above	799.05	0.00	2,726.06
**ALD**	South West	60-69	460.00	15.44	904.56
**ALD**	South West	70-79	824.45	38.56	1,610.33
**ALD**	South West	50-59	563.89	88.98	1,038.80
**ALD**	South West	30-39	NA	NA	NA
**ALD**	South Central	40-49	225.39	0.00	693.98
**ALD**	South Central	50-59	417.87	0.00	872.55
**ALD**	South Central	80 and above	742.90	0.00	2,411.25
**ALD**	South Central	70-79	815.40	0.00	1,695.51
**ALD**	South Central	60-69	744.23	122.22	1,366.23
**ALD**	South Central	30-39	NA	NA	NA
**ALD**	London	40-49	272.93	0.00	689.22
**ALD**	London	80 and above	738.85	0.00	2,297.48
**ALD**	London	50-59	457.84	62.02	853.65
**ALD**	London	70-79	1,074.01	116.36	2,031.66
**ALD**	London	60-69	601.80	129.27	1,074.33
**ALD**	London	30-39	NA	NA	NA
**ALD**	South East Coast	40-49	181.62	0.00	559.31
**ALD**	South East Coast	80 and above	495.25	0.00	1,795.64
**ALD**	South East Coast	70-79	941.57	6.38	1,876.76
**ALD**	South East Coast	50-59	535.94	14.67	1,057.20
**ALD**	South East Coast	60-69	778.32	140.09	1,416.54
**ALD**	South East Coast	30-39	NA	NA	NA
**ALD**	England	30-39	132.18	0.00	336.36
**ALD**	England	40-49	227.74	90.24	365.24
**ALD**	England	80 and above	762.86	152.11	1,373.61
**ALD**	England	50-59	473.67	321.45	625.88
**ALD**	England	60-69	628.20	442.01	814.38
**ALD**	England	70-79	948.64	627.11	1,270.16
**Autoimmune liver disease**	North East	70-79	1,065.04	0.00	3,992.19
**Autoimmune liver disease**	North East	80 and above	1,327.30	0.00	6,474.91
**Autoimmune liver disease**	North East	40-49	NA	NA	NA
**Autoimmune liver disease**	North East	50-59	NA	NA	NA
**Autoimmune liver disease**	North East	60-69	NA	NA	NA
**Autoimmune liver disease**	North West	50-59	505.55	0.00	1,523.63
**Autoimmune liver disease**	North West	60-69	513.14	0.00	1,371.87
**Autoimmune liver disease**	North West	80 and above	546.52	0.00	1,584.84
**Autoimmune liver disease**	North West	70-79	921.34	0.00	2,018.61
**Autoimmune liver disease**	North West	30-39	NA	NA	NA
**Autoimmune liver disease**	North West	40-49	NA	NA	NA
**Autoimmune liver disease**	Yorkshire & The Humber	30-39	NA	NA	NA
**Autoimmune liver disease**	Yorkshire & The Humber	40-49	NA	NA	NA
**Autoimmune liver disease**	Yorkshire & The Humber	50-59	NA	NA	NA
**Autoimmune liver disease**	Yorkshire & The Humber	60-69	NA	NA	NA
**Autoimmune liver disease**	Yorkshire & The Humber	70-79	NA	NA	NA
**Autoimmune liver disease**	Yorkshire & The Humber	80 and above	NA	NA	NA
**Autoimmune liver disease**	East Midlands	70-79	1,549.45	0.00	5,228.39
**Autoimmune liver disease**	East Midlands	30-39	NA	NA	NA
**Autoimmune liver disease**	East Midlands	40-49	NA	NA	NA
**Autoimmune liver disease**	East Midlands	50-59	NA	NA	NA
**Autoimmune liver disease**	East Midlands	60-69	NA	NA	NA
**Autoimmune liver disease**	East Midlands	80 and above	NA	NA	NA
**Autoimmune liver disease**	West Midlands	60-69	286.67	0.00	993.61
**Autoimmune liver disease**	West Midlands	80 and above	382.72	0.00	1,744.14
**Autoimmune liver disease**	West Midlands	70-79	445.30	0.00	1,447.71
**Autoimmune liver disease**	West Midlands	30-39	NA	NA	NA
**Autoimmune liver disease**	West Midlands	40-49	NA	NA	NA
**Autoimmune liver disease**	West Midlands	50-59	NA	NA	NA
**Autoimmune liver disease**	East of England	70-79	268.44	0.00	1,009.17
**Autoimmune liver disease**	East of England	60-69	406.77	0.00	1,409.29
**Autoimmune liver disease**	East of England	80 and above	777.33	0.00	2,416.82
**Autoimmune liver disease**	East of England	30-39	NA	NA	NA
**Autoimmune liver disease**	East of England	40-49	NA	NA	NA
**Autoimmune liver disease**	East of England	50-59	NA	NA	NA
**Autoimmune liver disease**	South West	60-69	320.75	0.00	1,187.09
**Autoimmune liver disease**	South West	70-79	354.67	0.00	1,153.42
**Autoimmune liver disease**	South West	50-59	471.37	0.00	1,594.72
**Autoimmune liver disease**	South West	80 and above	828.11	0.00	2,574.25
**Autoimmune liver disease**	South West	30-39	NA	NA	NA
**Autoimmune liver disease**	South West	40-49	NA	NA	NA
**Autoimmune liver disease**	South Central	70-79	300.64	0.00	1,068.58
**Autoimmune liver disease**	South Central	80 and above	476.28	0.00	1,942.86
**Autoimmune liver disease**	South Central	60-69	477.33	0.00	1,437.55
**Autoimmune liver disease**	South Central	30-39	NA	NA	NA
**Autoimmune liver disease**	South Central	40-49	NA	NA	NA
**Autoimmune liver disease**	South Central	50-59	NA	NA	NA
**Autoimmune liver disease**	London	60-69	203.82	0.00	754.66
**Autoimmune liver disease**	London	80 and above	854.74	0.00	3,094.99
**Autoimmune liver disease**	London	70-79	1,031.58	0.00	2,547.31
**Autoimmune liver disease**	London	30-39	NA	NA	NA
**Autoimmune liver disease**	London	40-49	NA	NA	NA
**Autoimmune liver disease**	London	50-59	NA	NA	NA
**Autoimmune liver disease**	South East Coast	80 and above	411.28	0.00	1,445.66
**Autoimmune liver disease**	South East Coast	60-69	428.28	0.00	1,245.83
**Autoimmune liver disease**	South East Coast	50-59	530.66	0.00	1,684.81
**Autoimmune liver disease**	South East Coast	70-79	580.19	0.00	1,666.15
**Autoimmune liver disease**	South East Coast	30-39	NA	NA	NA
**Autoimmune liver disease**	South East Coast	40-49	NA	NA	NA
**Autoimmune liver disease**	England	40-49	124.08	0.00	382.17
**Autoimmune liver disease**	England	50-59	338.95	8.95	668.94
**Autoimmune liver disease**	England	80 and above	580.29	72.61	1,087.98
**Autoimmune liver disease**	England	60-69	386.83	90.21	683.44
**Autoimmune liver disease**	England	70-79	602.94	221.89	984.00
**Autoimmune liver disease**	England	30-39	NA	NA	NA
**HBV**	North East	30-39	NA	NA	NA
**HBV**	North East	40-49	NA	NA	NA
**HBV**	North East	50-59	NA	NA	NA
**HBV**	North East	60-69	NA	NA	NA
**HBV**	North East	70-79	NA	NA	NA
**HBV**	North West	40-49	126.71	0.00	411.38
**HBV**	North West	60-69	227.70	0.00	789.41
**HBV**	North West	50-59	267.79	0.00	680.16
**HBV**	North West	70-79	405.06	0.00	1,317.05
**HBV**	North West	80 and above	605.73	0.00	2,963.49
**HBV**	North West	30-39	NA	NA	NA
**HBV**	Yorkshire & The Humber	30-39	NA	NA	NA
**HBV**	Yorkshire & The Humber	40-49	NA	NA	NA
**HBV**	Yorkshire & The Humber	50-59	NA	NA	NA
**HBV**	Yorkshire & The Humber	60-69	NA	NA	NA
**HBV**	Yorkshire & The Humber	70-79	NA	NA	NA
**HBV**	Yorkshire & The Humber	80 and above	NA	NA	NA
**HBV**	East Midlands	30-39	NA	NA	NA
**HBV**	East Midlands	40-49	NA	NA	NA
**HBV**	East Midlands	50-59	NA	NA	NA
**HBV**	East Midlands	60-69	NA	NA	NA
**HBV**	East Midlands	70-79	NA	NA	NA
**HBV**	East Midlands	80 and above	NA	NA	NA
**HBV**	West Midlands	50-59	282.69	0.00	957.03
**HBV**	West Midlands	60-69	305.12	0.00	1,057.52
**HBV**	West Midlands	70-79	503.39	0.00	1,890.81
**HBV**	West Midlands	30-39	NA	NA	NA
**HBV**	West Midlands	40-49	NA	NA	NA
**HBV**	West Midlands	80 and above	NA	NA	NA
**HBV**	East of England	60-69	273.94	0.00	1,014.04
**HBV**	East of England	30-39	NA	NA	NA
**HBV**	East of England	40-49	NA	NA	NA
**HBV**	East of England	50-59	NA	NA	NA
**HBV**	East of England	70-79	NA	NA	NA
**HBV**	East of England	80 and above	NA	NA	NA
**HBV**	South West	60-69	269.83	0.00	846.14
**HBV**	South West	50-59	333.37	0.00	961.90
**HBV**	South West	30-39	NA	NA	NA
**HBV**	South West	40-49	NA	NA	NA
**HBV**	South West	70-79	NA	NA	NA
**HBV**	South West	80 and above	NA	NA	NA
**HBV**	South Central	60-69	313.72	0.00	983.65
**HBV**	South Central	30-39	NA	NA	NA
**HBV**	South Central	40-49	NA	NA	NA
**HBV**	South Central	50-59	NA	NA	NA
**HBV**	South Central	70-79	NA	NA	NA
**HBV**	South Central	80 and above	NA	NA	NA
**HBV**	London	50-59	225.14	0.00	571.91
**HBV**	London	60-69	390.44	0.00	1,020.46
**HBV**	London	80 and above	449.93	0.00	1,931.24
**HBV**	London	70-79	519.15	0.00	1,627.43
**HBV**	London	30-39	NA	NA	NA
**HBV**	London	40-49	NA	NA	NA
**HBV**	South East Coast	30-39	NA	NA	NA
**HBV**	South East Coast	40-49	NA	NA	NA
**HBV**	South East Coast	50-59	NA	NA	NA
**HBV**	South East Coast	60-69	NA	NA	NA
**HBV**	South East Coast	70-79	NA	NA	NA
**HBV**	South East Coast	80 and above	NA	NA	NA
**HBV**	England	30-39	52.82	0.00	168.26
**HBV**	England	40-49	101.25	0.00	222.86
**HBV**	England	70-79	365.65	0.00	742.37
**HBV**	England	80 and above	534.91	0.00	1,415.08
**HBV**	England	60-69	275.90	49.09	502.71
**HBV**	England	50-59	219.50	50.45	388.55
**HCV**	North East	50-59	1,510.35	0.00	4,536.54
**HCV**	North East	30-39	NA	NA	NA
**HCV**	North East	40-49	NA	NA	NA
**HCV**	North East	60-69	NA	NA	NA
**HCV**	North East	70-79	NA	NA	NA
**HCV**	North East	80 and above	NA	NA	NA
**HCV**	North West	30-39	126.38	0.00	428.81
**HCV**	North West	40-49	127.43	0.00	321.92
**HCV**	North West	50-59	250.37	0.00	541.84
**HCV**	North West	60-69	283.28	0.00	888.31
**HCV**	North West	70-79	970.91	0.00	3,442.58
**HCV**	North West	80 and above	NA	NA	NA
**HCV**	Yorkshire & The Humber	30-39	NA	NA	NA
**HCV**	Yorkshire & The Humber	40-49	NA	NA	NA
**HCV**	Yorkshire & The Humber	50-59	NA	NA	NA
**HCV**	Yorkshire & The Humber	60-69	NA	NA	NA
**HCV**	Yorkshire & The Humber	70-79	NA	NA	NA
**HCV**	Yorkshire & The Humber	80 and above	NA	NA	NA
**HCV**	East Midlands	30-39	NA	NA	NA
**HCV**	East Midlands	40-49	NA	NA	NA
**HCV**	East Midlands	50-59	NA	NA	NA
**HCV**	East Midlands	60-69	NA	NA	NA
**HCV**	East Midlands	70-79	NA	NA	NA
**HCV**	East Midlands	80 and above	NA	NA	NA
**HCV**	West Midlands	40-49	199.48	0.00	690.27
**HCV**	West Midlands	50-59	417.02	0.00	1,202.93
**HCV**	West Midlands	60-69	693.34	0.00	2,086.58
**HCV**	West Midlands	30-39	NA	NA	NA
**HCV**	West Midlands	70-79	NA	NA	NA
**HCV**	West Midlands	80 and above	NA	NA	NA
**HCV**	East of England	40-49	146.61	0.00	507.39
**HCV**	East of England	50-59	225.89	0.00	652.02
**HCV**	East of England	60-69	429.79	0.00	1,178.72
**HCV**	East of England	30-39	NA	NA	NA
**HCV**	East of England	70-79	NA	NA	NA
**HCV**	East of England	80 and above	NA	NA	NA
**HCV**	South West	40-49	102.09	0.00	353.37
**HCV**	South West	30-39	247.60	0.00	839.76
**HCV**	South West	50-59	362.36	0.00	817.75
**HCV**	South West	60-69	404.38	0.00	1,056.85
**HCV**	South West	70-79	NA	NA	NA
**HCV**	South West	80 and above	NA	NA	NA
**HCV**	South Central	40-49	207.89	0.00	674.77
**HCV**	South Central	50-59	235.25	0.00	679.00
**HCV**	South Central	60-69	609.17	0.00	1,500.20
**HCV**	South Central	30-39	NA	NA	NA
**HCV**	South Central	70-79	NA	NA	NA
**HCV**	South Central	80 and above	NA	NA	NA
**HCV**	London	40-49	175.63	0.00	497.73
**HCV**	London	50-59	310.12	0.00	654.99
**HCV**	London	60-69	438.71	0.00	990.88
**HCV**	London	70-79	535.69	0.00	1,538.58
**HCV**	London	30-39	NA	NA	NA
**HCV**	London	80 and above	NA	NA	NA
**HCV**	South East Coast	50-59	288.27	0.00	869.43
**HCV**	South East Coast	70-79	623.87	0.00	2,506.33
**HCV**	South East Coast	30-39	NA	NA	NA
**HCV**	South East Coast	40-49	NA	NA	NA
**HCV**	South East Coast	60-69	NA	NA	NA
**HCV**	South East Coast	80 and above	NA	NA	NA
**HCV**	England	30-39	142.79	0.00	318.06
**HCV**	England	80 and above	447.56	0.00	1,489.48
**HCV**	England	70-79	532.15	0.00	1,114.87
**HCV**	England	40-49	155.84	38.05	273.64
**HCV**	England	50-59	297.98	142.88	453.08
**HCV**	England	60-69	423.28	155.43	691.14
**NAFLD**	North East	60-69	336.76	0.00	979.90
**NAFLD**	North East	50-59	651.46	0.00	1,613.45
**NAFLD**	North East	80 and above	748.13	0.00	3,207.51
**NAFLD**	North East	70-79	879.43	0.00	2,463.24
**NAFLD**	North East	30-39	NA	NA	NA
**NAFLD**	North East	40-49	NA	NA	NA
**NAFLD**	North West	40-49	196.94	0.00	422.85
**NAFLD**	North West	80 and above	872.66	0.00	1,886.27
**NAFLD**	North West	50-59	447.46	164.44	730.48
**NAFLD**	North West	60-69	615.81	263.31	968.31
**NAFLD**	North West	70-79	955.26	349.95	1,560.57
**NAFLD**	North West	30-39	NA	NA	NA
**NAFLD**	Yorkshire & The Humber	60-69	416.23	0.00	1,087.78
**NAFLD**	Yorkshire & The Humber	50-59	619.36	0.00	1,419.27
**NAFLD**	Yorkshire & The Humber	80 and above	646.51	0.00	2,943.27
**NAFLD**	Yorkshire & The Humber	70-79	795.82	0.00	1,966.54
**NAFLD**	Yorkshire & The Humber	30-39	NA	NA	NA
**NAFLD**	Yorkshire & The Humber	40-49	NA	NA	NA
**NAFLD**	East Midlands	50-59	506.09	0.00	1,253.95
**NAFLD**	East Midlands	60-69	508.97	0.00	1,329.77
**NAFLD**	East Midlands	70-79	728.70	0.00	1,920.54
**NAFLD**	East Midlands	80 and above	1,282.35	0.00	4,367.36
**NAFLD**	East Midlands	30-39	NA	NA	NA
**NAFLD**	East Midlands	40-49	NA	NA	NA
**NAFLD**	West Midlands	40-49	218.24	0.00	597.87
**NAFLD**	West Midlands	80 and above	607.34	0.00	1,820.05
**NAFLD**	West Midlands	60-69	509.27	10.78	1,007.76
**NAFLD**	West Midlands	50-59	479.74	27.82	931.65
**NAFLD**	West Midlands	70-79	864.12	64.23	1,664.01
**NAFLD**	West Midlands	30-39	NA	NA	NA
**NAFLD**	East of England	40-49	203.45	0.00	557.38
**NAFLD**	East of England	50-59	358.09	0.00	725.54
**NAFLD**	East of England	80 and above	800.26	0.00	1,960.01
**NAFLD**	East of England	70-79	760.50	20.38	1,500.61
**NAFLD**	East of England	60-69	639.82	99.58	1,180.06
**NAFLD**	East of England	30-39	NA	NA	NA
**NAFLD**	South West	40-49	119.54	0.00	352.15
**NAFLD**	South West	30-39	245.20	0.00	831.63
**NAFLD**	South West	80 and above	1,050.43	0.00	2,327.78
**NAFLD**	South West	50-59	407.60	72.33	742.88
**NAFLD**	South West	70-79	724.20	93.70	1,354.70
**NAFLD**	South West	60-69	489.95	117.45	862.45
**NAFLD**	South Central	40-49	161.04	0.00	441.24
**NAFLD**	South Central	50-59	314.71	0.00	637.73
**NAFLD**	South Central	80 and above	905.06	0.00	2,198.15
**NAFLD**	South Central	70-79	727.45	40.52	1,414.37
**NAFLD**	South Central	60-69	599.38	111.94	1,086.83
**NAFLD**	South Central	30-39	NA	NA	NA
**NAFLD**	London	40-49	210.51	0.00	457.39
**NAFLD**	London	80 and above	786.58	0.00	1,868.08
**NAFLD**	London	50-59	348.79	88.36	609.23
**NAFLD**	London	60-69	478.22	135.71	820.73
**NAFLD**	London	70-79	916.98	234.75	1,599.21
**NAFLD**	London	30-39	NA	NA	NA
**NAFLD**	South East Coast	40-49	201.32	0.00	508.50
**NAFLD**	South East Coast	50-59	318.70	0.00	639.90
**NAFLD**	South East Coast	80 and above	828.40	0.00	1,981.66
**NAFLD**	South East Coast	60-69	538.71	100.47	976.96
**NAFLD**	South East Coast	70-79	713.18	125.57	1,300.78
**NAFLD**	South East Coast	30-39	NA	NA	NA
**NAFLD**	England	30-39	76.31	0.00	178.42
**NAFLD**	England	40-49	192.67	90.20	295.14
**NAFLD**	England	50-59	402.87	283.00	522.74
**NAFLD**	England	60-69	537.75	389.98	685.53
**NAFLD**	England	80 and above	852.54	427.49	1,277.60
**NAFLD**	England	70-79	815.87	576.08	1,055.66
