# Supplementary material for: Increased burden of cardiovascular disease in people with liver disease: unequal geographical variations, risk factors and excess years of life lost
Source: J Transl Med. 2022 Jan 3;20:2. doi: 10.1186/s12967-021-03210-9 (PMC8722174; doi:10.1186/s12967-021-03210-9)
Supplement: Supplementary file 10 — Additional file 10: Age-specific incidence rates for cardiovascular disease in patients without liver disease. [file 12967_2021_3210_MOESM10_ESM.pdf]

Additional file 10. Age-specific incidence rates for cardiovascular disease in patients without liver disease.

Practice region	Age group	Incidence rate (per 100,000 person years)	Lower CI	Upper CI
North East	30-39	13.35	0.71	25.99
North West	30-39	19.05	13.65	24.45
Yorkshire & The Humber	30-39	17.12	4.41	29.83
East Midlands	30-39	15.43	1.05	29.81
West Midlands	30-39	17.89	11.72	24.06
East of England	30-39	16.36	10.10	22.62
South West	30-39	15.88	10.14	21.63
South Central	30-39	13.36	8.45	18.28
London	30-39	11.70	7.93	15.47
South East Coast	30-39	16.39	11.37	21.41
England	30-39	15.56	13.66	17.46
North East	40-49	49.60	28.15	71.06
North West	40-49	53.44	45.38	61.49
Yorkshire & The Humber	40-49	42.02	28.46	55.59
East Midlands	40-49	43.88	29.47	58.30
West Midlands	40-49	44.50	36.08	52.91
East of England	40-49	38.75	31.11	46.39
South West	40-49	41.09	33.24	48.93
South Central	40-49	36.05	29.10	43.00
London	40-49	35.44	29.27	41.61
South East Coast	40-49	40.49	33.41	47.56
England	40-49	41.60	38.92	44.27
North East	50-59	160.50	123.32	197.67
North West	50-59	148.10	134.81	161.38
Yorkshire & The Humber	50-59	111.06	90.29	131.83
East Midlands	50-59	111.57	89.28	133.86
West Midlands	50-59	122.78	108.79	136.77
East of England	50-59	109.63	96.65	122.61
South West	50-59	125.79	112.59	138.98
South Central	50-59	106.76	94.82	118.70
London	50-59	107.13	95.26	119.00
South East Coast	50-59	112.46	100.76	124.16
England	50-59	119.54	114.99	124.09
North East	60-69	251.23	197.83	304.63
North West	60-69	262.09	241.32	282.86
Yorkshire & The Humber	60-69	201.13	169.88	232.37
East Midlands	60-69	214.97	178.17	251.78
West Midlands	60-69	221.28	199.77	242.79
East of England	60-69	192.41	172.24	212.58
South West	60-69	209.82	190.49	229.15
South Central	60-69	197.62	178.83	216.40
London	60-69	208.52	187.32	229.72
South East Coast	60-69	212.53	193.83	231.23
England	60-69	215.97	208.80	223.14
North East	70-79	418.45	331.89	505.02
North West	70-79	446.69	413.45	479.93
Yorkshire & The Humber	70-79	382.98	328.73	437.24
East Midlands	70-79	386.09	325.62	446.57
West Midlands	70-79	386.46	352.74	420.18
East of England	70-79	352.11	318.61	385.61
South West	70-79	383.64	352.59	414.69
South Central	70-79	354.87	324.23	385.51
London	70-79	375.03	337.66	412.40
South East Coast	70-79	375.68	345.80	405.55
England	70-79	384.39	372.71	396.08
North East	80 and above	561.48	430.18	692.78
North West	80 and above	607.17	553.58	660.76
Yorkshire & The Humber	80 and above	547.74	461.21	634.28
East Midlands	80 and above	566.52	469.69	663.35
West Midlands	80 and above	569.00	514.63	623.37
East of England	80 and above	537.62	484.18	591.07
South West	80 and above	557.60	508.56	606.63
South Central	80 and above	549.98	498.69	601.27
London	80 and above	552.98	494.52	611.43
South East Coast	80 and above	563.73	515.47	611.99
England	80 and above	562.59	543.85	581.32
