# Supplementary material for: Increased burden of cardiovascular disease in people with liver disease: unequal geographical variations, risk factors and excess years of life lost
Source: J Transl Med. 2022 Jan 3;20:2. doi: 10.1186/s12967-021-03210-9 (PMC8722174; doi:10.1186/s12967-021-03210-9)
Supplement: Supplementary file 11 — Additional file 11: Factors associated with the initial presentation of cardiovascular disease in individuals with liver disease. [file 12967_2021_3210_MOESM11_ESM.pdf]

Additional file 11. Factors associated with the initial presentation of cardiovascular disease in individuals with liver disease

	ALD		Autoimmune liver disease		HBV		HCV		NAFLD	
**Characteristic**	**Fully adjusted HR**	**P value**	**Fully adjusted HR**	**P value**	**Fully adjusted HR**	**P value**	**Fully adjusted HR**	**P value**	**Fully adjusted HR**	**P value**
Female	0.72 (0.67 - 0.78)	< 0.001	0.53 (0.45 - 0.64)	< 0.001	0.67 (0.52 - 0.87)	0.003	0.73 (0.6 - 0.88)	0.001	0.71 (0.67 - 0.76)	< 0.001
**Age**										
Age 30 - 39 (reference)	1.00 (ref)		1.00 (ref)		1.00 (ref)		1.00 (ref)		1.00 (ref)	
Age 40 - 49	1.6 (1.33 - 1.92)	< 0.001	3.27 (1.47 - 7.25)	0.004	2.67 (1.63 - 4.39)	< 0.001	1.49 (1.14 - 1.94)	0.003	2.2 (1.8 - 2.7)	< 0.001
Age 50 - 59	2.93 (2.47 - 3.47)	< 0.001	4.77 (2.21 - 10.29)	< 0.001	5.44 (3.4 - 8.71)	< 0.001	2.56 (1.96 - 3.36)	< 0.001	3.79 (3.12 - 4.6)	< 0.001
Age 60 - 69	4.98 (4.2 - 5.91)	< 0.001	9.33 (4.38 - 19.85)	< 0.001	11.29 (6.89 - 18.49)	< 0.001	4.67 (3.46 - 6.29)	< 0.001	6.87 (5.67 - 8.32)	< 0.001
Age 70 - 79	8.13 (6.75 - 9.79)	< 0.001	18.43 (8.67 - 39.16)	< 0.001	24.15 (13.85 - 42.11)	< 0.001	8.83 (6.08 - 12.84)	< 0.001	12.67 (10.43 - 15.39)	< 0.001
Age 80 & above	16.13 (12.67 - 20.53)	< 0.001	36.9 (17.09 - 79.68)	< 0.001	38.53 (19.6 - 75.76)	< 0.001	17.41 (10.5 - 28.87)	< 0.001	22.1 (17.96 - 27.19)	< 0.001
BMI ≥ 30 kg/m2	1.2 (1.11 - 1.3)	< 0.001	1.4 (1.17 - 1.67)	< 0.001	1.5 (1.12 - 2)	0.006	1.3 (1.03 - 1.64)	0.028	1.22 (1.15 - 1.3)	< 0.001
**Smoking**										
Non smoker (reference)	1.00 (ref)		1.00 (ref)		1.00 (ref)		1.00 (ref)		1.00 (ref)	
Current smoker	1.3 (1.18 - 1.43)	< 0.001	1.78 (1.45 - 2.18)	< 0.001	1.74 (1.25 - 2.43)	0.001	1.23 (0.95 - 1.6)	0.108	1.77 (1.64 - 1.91)	< 0.001
Ex smoker	1.12 (1.01 - 1.24)	0.034	1.45 (1.2 - 1.74)	< 0.001	1.35 (0.97 - 1.86)	0.074	1.06 (0.79 - 1.41)	0.707	1.32 (1.23 - 1.42)	< 0.001
Any smoker	1.01 (0.38 - 2.69)	0.989	11.14 (3.55 - 34.96)	< 0.001					1.49 (0.62 - 3.59)	0.373
No smoking information	1.15 (0.97 - 1.37)	0.097	1.34 (0.9 - 1.99)	0.149	0.95 (0.57 - 1.59)	0.847	0.94 (0.6 - 1.46)	0.772	1.47 (1.1 - 1.95)	0.009
**Comorbidities prior to cohort entry**										
Barrett's oesophagus	1.19 (0.9 - 1.59)	0.226	0.73 (0.35 - 1.55)	0.417	0.85 (0.12 - 6.09)	0.872	0.96 (0.36 - 2.59)	0.942	1.38 (1.12 - 1.7)	0.002
Crohn's disease	0.69 (0.36 - 1.33)	0.27	0.67 (0.33 - 1.35)	0.261	1.68 (0.53 - 5.32)	0.375	1.26 (0.41 - 3.94)	0.686	0.94 (0.69 - 1.29)	0.716
Diverticular disease of intestine	1.02 (0.86 - 1.2)	0.832	1.37 (1.07 - 1.76)	0.012	0.72 (0.36 - 1.42)	0.343	1.61 (0.94 - 2.76)	0.086	1.17 (1.06 - 1.29)	0.002
Diabetes mellitus	1.52 (1.38 - 1.66)	< 0.001	1.63 (1.34 - 1.98)	< 0.001	1.82 (1.32 - 2.53)	< 0.001	1.87 (1.47 - 2.38)	< 0.001	1.59 (1.49 - 1.69)	< 0.001
Complications of diabetes	1.62 (1.37 - 1.92)	< 0.001	1.63 (1.19 - 2.25)	0.002	1.5 (0.83 - 2.71)	0.178	2.8 (1.98 - 3.97)	< 0.001	1.73 (1.58 - 1.9)	< 0.001
Dyslipidaemia	1.22 (1.11 - 1.34)	< 0.001	1.09 (0.88 - 1.36)	0.437	1.27 (0.88 - 1.84)	0.197	1.86 (1.32 - 2.6)	< 0.001	1.25 (1.17 - 1.34)	< 0.001
Gastro-oesophageal reflux disease	1.09 (0.98 - 1.22)	0.107	1.32 (1.06 - 1.64)	0.013	1.01 (0.66 - 1.55)	0.957	0.99 (0.74 - 1.34)	0.97	1.17 (1.09 - 1.26)	< 0.001
Hypertension	1.34 (1.25 - 1.44)	< 0.001	1.29 (1.1 - 1.52)	0.002	2.1 (1.59 - 2.77)	< 0.001	1.69 (1.36 - 2.11)	< 0.001	1.46 (1.37 - 1.56)	< 0.001
Irritable bowel syndrome	1.02 (0.88 - 1.2)	0.768	0.93 (0.71 - 1.22)	0.605	0.51 (0.26 - 1)	0.049	0.77 (0.51 - 1.16)	0.208	0.97 (0.88 - 1.07)	0.58
Jaundice	1.05 (0.92 - 1.2)	0.467	0.83 (0.65 - 1.06)	0.132	0.98 (0.63 - 1.53)	0.925	1.49 (1.01 - 2.22)	0.047	1.08 (0.91 - 1.29)	0.366
Proteinuric kidney diseases	1.62 (1.16 - 2.25)	0.004	2.84 (1.67 - 4.83)	< 0.001	2.88 (1.56 - 5.3)	0.001	2.32 (1.42 - 3.79)	0.001	1.73 (1.42 - 2.1)	< 0.001
Oesophagitis and oesophageal ulcer	1.21 (1.08 - 1.35)	0.001	1.46 (1.14 - 1.86)	0.003	0.59 (0.34 - 1.04)	0.069	1.14 (0.8 - 1.61)	0.469	1.22 (1.12 - 1.33)	< 0.001
Proteinuria	1.1 (1.02 - 1.18)	0.009	1.14 (0.97 - 1.33)	0.108	1.66 (1.27 - 2.16)	< 0.001	1.18 (0.98 - 1.42)	0.087	1.19 (1.12 - 1.27)	< 0.001
Renal disease	1.39 (1.22 - 1.6)	< 0.001	1.69 (1.37 - 2.08)	< 0.001	2.12 (1.46 - 3.09)	< 0.001	2.03 (1.49 - 2.77)	< 0.001	1.44 (1.33 - 1.56)	< 0.001
**Biomarkers at cohort entry**										
Albumin < 35 g/L	1.2 (1.1 - 1.32)	< 0.001	1.55 (1.26 - 1.91)	< 0.001	2.39 (1.54 - 3.71)	< 0.001	1.75 (1.29 - 2.39)	< 0.001	1.59 (1.42 - 1.79)	< 0.001
Alanine aminotransferase ≥ 35 U/L	0.85 (0.78 - 0.93)	0.001	0.95 (0.8 - 1.14)	0.609	0.96 (0.65 - 1.44)	0.858	0.85 (0.67 - 1.07)	0.163	0.82 (0.75 - 0.9)	< 0.001
Aspartate transaminase ≥ 40 U/L	1.01 (0.89 - 1.15)	0.862	1.05 (0.8 - 1.37)	0.712	1.11 (0.62 - 2)	0.723	0.75 (0.5 - 1.15)	0.186	0.97 (0.83 - 1.15)	0.76
Bilirubin ≥ 34.2 μmol/L	1.04 (0.93 - 1.16)	0.485	1.23 (0.94 - 1.6)	0.137	1.46 (0.87 - 2.43)	0.149	1.31 (0.82 - 2.1)	0.261	1.11 (0.92 - 1.32)	0.279
C reactive protein ≥ 10 mg/L	1.26 (1.09 - 1.44)	0.001	1.15 (0.89 - 1.48)	0.28	0.74 (0.33 - 1.67)	0.463	1.11 (0.67 - 1.83)	0.679	1.32 (1.18 - 1.47)	< 0.001
Gamma- glutamyltransferase ≥ 48 U/L	0.96 (0.89 - 1.04)	0.35	1.01 (0.85 - 1.2)	0.936	1.64 (1.06 - 2.55)	0.028	0.83 (0.61 - 1.13)	0.246	1.04 (0.95 - 1.13)	0.438
International Normalized Ratio ≥ 1.7	1.31 (0.94 - 1.83)	0.113	3.49 (2.11 - 5.79)	< 0.001	2.46 (1 - 6.06)	0.05	6.43 (3.18 - 13.03)	< 0.001	1.46 (1.18 - 1.81)	< 0.001
Platelet ≤ 150 10^9/L	1.18 (1.08 - 1.29)	< 0.001	1.25 (0.96 - 1.63)	0.095	1.59 (1.05 - 2.39)	0.028	1.17 (0.89 - 1.53)	0.254	1.48 (1.32 - 1.67)	< 0.001
Triglycerides ≥ 2 mmol/L	0.94 (0.83 - 1.07)	0.37	1.13 (0.8 - 1.62)	0.485	1.03 (0.51 - 2.08)	0.943	1.55 (1 - 2.41)	0.051	1.19 (1.1 - 1.29)	< 0.001
