# Supplementary material for: Increased burden of cardiovascular disease in people with liver disease: unequal geographical variations, risk factors and excess years of life lost
Source: J Transl Med. 2022 Jan 3;20:2. doi: 10.1186/s12967-021-03210-9 (PMC8722174; doi:10.1186/s12967-021-03210-9)
Supplement: Supplementary file 12 — Additional file 12: Sensitivity analyses on complete records. [file 12967_2021_3210_MOESM12_ESM.pdf]

Additional file 12. Sensitivity analyses on complete records

Characteristic	Adjusted HR	P value	Liver disease type
BMI ≥ 30 kg/m2	1.21 (1.12 - 1.32)	< 0.001	ALD
BMI ≥ 30 kg/m2	1.37 (1.14 - 1.65)	0.001	Autoimmune liver disease
BMI ≥ 30 kg/m2	1.52 (1.13 - 2.05)	0.006	HBV
BMI ≥ 30 kg/m2	1.31 (1.03 - 1.66)	0.027	HCV
BMI ≥ 30 kg/m2	1.24 (1.16 - 1.31)	< 0.001	NAFLD
Alanine aminotransferase ≥ 35 U/L	0.73 (0.64 - 0.84)	< 0.001	ALD
Alanine aminotransferase ≥ 35 U/L	0.68 (0.52 - 0.9)	0.008	Autoimmune liver disease
Alanine aminotransferase ≥ 35 U/L	0.82 (0.47 - 1.44)	0.496	HBV
Alanine aminotransferase ≥ 35 U/L	0.88 (0.58 - 1.33)	0.537	HCV
Alanine aminotransferase ≥ 35 U/L	0.73 (0.65 - 0.83)	< 0.001	NAFLD
Albumin < 35 g/L	1.26 (1.14 - 1.39)	< 0.001	ALD
Albumin < 35 g/L	1.57 (1.26 - 1.96)	< 0.001	Autoimmune liver disease
Albumin < 35 g/L	2.14 (1.32 - 3.46)	0.002	HBV
Albumin < 35 g/L	1.99 (1.42 - 2.78)	< 0.001	HCV
Albumin < 35 g/L	1.6 (1.42 - 1.8)	< 0.001	NAFLD
C reactive protein ≥ 10 mg/L	1.47 (1.22 - 1.77)	< 0.001	ALD
C reactive protein ≥ 10 mg/L	1.34 (0.97 - 1.85)	0.074	Autoimmune liver disease
C reactive protein ≥ 10 mg/L	0.78 (0.28 - 2.2)	0.638	HBV
C reactive protein ≥ 10 mg/L	1.36 (0.76 - 2.43)	0.306	HCV
C reactive protein ≥ 10 mg/L	1.45 (1.26 - 1.65)	< 0.001	NAFLD
International Normalized Ratio ≥ 1.7	1.28 (0.84 - 1.95)	0.25	ALD
International Normalized Ratio ≥ 1.7	4.14 (1.98 - 8.67)	< 0.001	Autoimmune liver disease
International Normalized Ratio ≥ 1.7	3.09 (0.75 - 12.68)	0.117	HBV
International Normalized Ratio ≥ 1.7	7.81 (2.22 - 27.48)	0.001	HCV
International Normalized Ratio ≥ 1.7	1.51 (1.02 - 2.23)	0.038	NAFLD
