# Supplementary material for: Increased burden of cardiovascular disease in people with liver disease: unequal geographical variations, risk factors and excess years of life lost
Source: J Transl Med. 2022 Jan 3;20:2. doi: 10.1186/s12967-021-03210-9 (PMC8722174; doi:10.1186/s12967-021-03210-9)
Supplement: Supplementary file 13 — Additional file 13. Supplementary methods and results. [file 12967_2021_3210_MOESM13_ESM.pdf]

Additional file 13: Supplementary methods and results

Regional variations in age-standardised and age-specific incidence rates for liver disease

An exploration of regional variations revealed that the patterns of incidence burden differed across liver diseases (Additional file 5). North West England had the highest age-standardised incidence rate for any liver disease (151 per 100,000 person years), while the lowest incidence rate was observed in Yorkshire and the Humber (87 per 100,000 person years) (Figure 1A, Additional file 5).

Delving into specific liver diseases, regions with the highest incidence rates were as follow: ALD (North West; 35 per 100,000 person years), autoimmune liver disease (North East; 6 per 100,000 person years), HBV (London; 11 per 100,000 person years), HCV (North East; 10 per 100,000 person years) and NAFLD (London; 116 per 100,000 person years). The North West and North East regions consistently exhibited relatively high age-standardised incidence rates for individual liver diseases (except for HBV), while the regions in the east of England (i.e., Yorkshire and The Humber, East Midlands and East of England), had consistently low incidence rates (Figure 1A, Additional file 5). For HBV, however, we found that London had a much higher incidence rate compared to all other regions (11 in London vs. 0.8 to 3.6 per 100,000 person years in other regions).

Regionally, the maximum incidence for any liver disease was reached in individuals aged 50-59 for all regions except the West Midlands (where incidence peaked in individuals age 60-69) (Additional file 6). Additional age-specific incidence rates by specific liver disease type and geographical regions were provided in Additional file 6.

Regional variations in age-standardised and age-specific incidence rates for CVD in patients with or without liver disease

When exploring regional differences in patients with liver disease, individuals from the North East (3,179 per 100,000 person years, CI: 2,423-3,935) and North West (2,960 per 100,000 person years, CI: 2,704-3,217) had the highest and second highest incidence burdens for CVD, respectively (Figure 2A, Additional file 7). Similarly, the North West region had the highest incidence for CVD in people without liver disease (1,537 per 100,000 person years) (Figure 2B, Additional file 8). In individuals with liver disease, the lowest CVD incidence was observed in the South East region (2,420 per 100,000 person years, CI: 2,098-2,742) (Figure 2A, Additional file 7). However, in people without liver disease, East of England had the lowest CVD incidence rate (1,226 per 100,000 person years) (Figure 2B, Additional file 8).

Variation rates were more striking when comparing liver disease types. Regions with the highest incidence burdens of CVD by liver disease type were as follow: ALD (North East; 3,827 per 100,000 person years), autoimmune (North East; 3,512 per 100,000 person years), HBV (West Midlands; 3,119 per 100,000 person years), HCV (South Central; 3,714 per 100,000 person years) and NAFLD (East Midlands; 3,325 per 100,000 person years) (Figure 2A, Additional file 7).

When investigating regional variations in age-specific incidence rates, the highest CVD incidence in people with and without liver disease was observed in individuals aged 70 and above across all regions (Additional file 9, Additional file 10).

Comorbidity patterns in patients with liver disease

We analysed comorbidity patterns for 14 prevalent conditions stratified by liver disease type, age group and sex (Additional file 1). Comorbidities were common in people with liver disease. A general trend of increased in diagnosed comorbidities was observed across all liver diseases. Men with autoimmune liver disease had an increased prevalence of Crohn's disease especially in the younger age groups: age 30-39 (8.3%) and age 40-49 (11.8%) (Additional file 1). The prevalence of Barrett's oesophagus, renal disease, diabetes mellitus, complications of diabetes, diverticular disease of the intestine, dyslipidaemia and hypertension exhibited an upward trend with increasing age and displayed consistent patterns across the five liver conditions. Among patients with HBV at ages 70-79, diabetes was present in 31.5% men and 29.0% women, whereas, in patients aged 40-49, the proportions were 7.2% and 6.5% in men and women respectively. Similarly, among patients with NAFLD, renal disease was present in 18.3% men and 23.5% women of ages 70-79, whereas, in patients aged 40-49, only 3.3% men and 8.6% women had renal disease (Additional file 1).

Liver disease phenotypes

NAFLD

Code	Description	Coding system	Data source
**J61y700**	Steatosis of liver	Read code v2	Primary care
**J61y900**	Fatty change of liver	Read code v2	Primary care
**J61y911**	Fatty liver	Read code v2	Primary care
**J61y100**	Nonalcoholic fatty liver disease	Read code v2	Primary care
**J61y800**	Nonalcoholic steatohepatitis	Read code v2	Primary care
**K76.0**	Fatty (change of) liver, not elsewhere classified	ICD-10	Secondary care

Alcoholic liver disease

Code	Description	Coding system	Data source
**G852300**	Oesophageal varices in alcoholic cirrhosis of the liver	Read code v2	Primary care
**J610.00**	Alcoholic fatty liver	Read code v2	Primary care
**J612.00**	Alcoholic cirrhosis of liver	Read code v2	Primary care
**J612.12**	Laennec's cirrhosis	Read code v2	Primary care
**J612000**	Alcoholic fibrosis and sclerosis of liver	Read code v2	Primary care
**J613.00**	Alcoholic liver damage unspecified	Read code v2	Primary care
**J613000**	Alcoholic hepatic failure	Read code v2	Primary care
**J617.00**	Alcoholic hepatitis	Read code v2	Primary care
**J617000**	Chronic alcoholic hepatitis	Read code v2	Primary care
**K70**	Alcoholic liver disease	ICD-10	Secondary care

Autoimmune liver disease

Code	Description	Coding system	Data source

**J614111**	Autoimmune chronic active hepatitis	Read code v2	Primary care
**J616000**	Previously known as primary biliary cirrhosis	Read code v2	Primary care
**J63B.00**	Autoimmune hepatitis	Read code v2	Primary care
**J661700**	Primary sclerosing cholangitis	Read code v2	Primary care
**K74.3**	Previously known as primary biliary cirrhosis	ICD-10	Secondary care
**K75.4**	Autoimmune hepatitis	ICD-10	Secondary care

Viral hepatitis

Code	Description	Coding system	Data source
**43B4.00**	Hepatitis B surface antigen +ve	Read code v2	Primary care
**43B5.00**	Hepatitis e antigen present	Read code v2	Primary care
**43X3.00**	Hepatitis C antibody test positive	Read code v2	Primary care
**43j5.00**	Hepatitis C nucleic acid detection	Read code v2	Primary care
**43jG.00**	Hepatitis B nucleic acid detection	Read code v2	Primary care
**4J3B.00**	Hepatitis C viral load	Read code v2	Primary care
**4J3D.00**	Hepatitis B viral load	Read code v2	Primary care
**4JQ3.00**	Hepatitis C virus genotype	Read code v2	Primary care
**4JQD.00**	Hepatitis C viral ribonucleic acid PCR positive	Read code v2	Primary care
**4JQD.11**	Hepatitis C PCR positive	Read code v2	Primary care
**4JQF.00**	Hepatitis C antigen positive	Read code v2	Primary care
**7Q05200**	Hepatitis B treatment drugs Band 1	Read code v2	Primary care
**9kV..00**	Hepatitis C screening positive - enhanced services admin	Read code v2	Primary care
**9kV..11**	Hepatitis C screening positive	Read code v2	Primary care
**9kZ..00**	Hepatitis B screening positive - enhanced services admin	Read code v2	Primary care
**9kZ..11**	Hepatitis B screening positive	Read code v2	Primary care
**A703.00**	Viral (serum) hepatitis B	Read code v2	Primary care
**A705000**	Viral hepatitis C without mention of hepatic coma	Read code v2	Primary care
**A705100**	Acute delta-(super)infection of hepatitis B carrier	Read code v2	Primary care
**A707000**	Chronic viral hepatitis B with delta-agent	Read code v2	Primary care
**A707100**	Chronic viral hepatitis B without delta-agent	Read code v2	Primary care
**A707200**	Chronic viral hepatitis C	Read code v2	Primary care
**A707300**	Chronic viral hepatitis B	Read code v2	Primary care
**A70A.00**	Hepatitis C genotype 1	Read code v2	Primary care
**A70B.00**	Hepatitis C genotype 2	Read code v2	Primary care
**A70C.00**	Hepatitis C genotype 3	Read code v2	Primary care
**A70D.00**	Hepatitis C genotype 4	Read code v2	Primary care
**A70z000**	Hepatitis C	Read code v2	Primary care
**Q409100**	Congenital hepatitis B infection	Read code v2	Primary care
**B18.0**	Chronic viral hepatitis B with delta-agent	ICD-10	Secondary care
**B18.1**	Chronic viral hepatitis B without delta-agent, Chronic (viral) hepatitis B	ICD-10	Secondary care
**B18.2**	Chronic viral hepatitis C	ICD-10	Secondary care
